# Palm Kernel Meal Inclusion Does Not Compromise Performance of Growing and Finishing Pigs Regardless of Feed Form or Corn Processing

**DOI:** 10.3390/vetsci13090963

**Published:** 2026-09-14

**Authors:** Vetriselvi Sampath, In Ho Kim

**Affiliations:** 1 Department of Animal Biotechnology, Dankook University, Cheonan 31116, Republic of Korea; vetri09@dankook.ac.kr; 2 Smart Animal Bio Institute, Dankook University, Cheonan 31116, Republic of Korea

**Keywords:** growth performance, nutrient digestibility, carcass quality, feed form, pigs

## Abstract

This study aims to assess the impact of different forms of feed and their processing methods on growth performance, nutrient absorption, and carcass quality in pigs across both growing and finishing stages. A total of 440 crossbred (Yorkshire × Landrace × Duroc) pigs were used in two experiments. Experiment 1 (Exp. 1) included 220 growing pigs (63 days of age; initial body weight [BW], 25.06 ± 1.82 kg), while Experiment 2 (Exp. 2) included 220 finishing pigs (105 days of age; initial BW, 54.80 ± 3.66 kg). In both experiments, pigs were assigned to four dietary treatments arranged in a 2 × 2 factorial design with 11 replicates with five pigs (two gilts and three barrows)/pen. In Exp. 1, growing pigs were either fed mash and/or crumble diets with or without 5% PKM for 6 weeks. Herein, corn, soybean meal (SBM), and PKM were all processed into both mash and crumble forms. In Exp. 2., finishing pigs were fed with mash (extruded) and crumble (non-extruded) diets with and without PKM for 10 weeks. At the end of week 6, growing pigs fed neither mash nor crumble with and without PKM diets showed no significant (*p* > 0.05) changes in their growth performance and nutrient digestibility. Similarly, no notable (*p* > 0.05) changes were observed on the growth performance, nutrient digestibility, and carcass grade quality in finishing pigs at the end of week 10. In summary, the present study had insufficient (less than 80%) statistical power to show possible differences between the treatment groups suggesting that current findings are preliminary evidence regarding the use of PKM and different feed-processing strategies in pig diets; however, further studies with greater experimental replication are warranted to validate these findings to clarify potential treatment effects.

## Simple Summary

Improving feed efficiency is essential for reducing production costs and supporting sustainable pig farming. Using appropriate feed-processing methods, such as crumbling and/or extrusion, together with cost-effective ingredients like palm kernel meal (PKM), could help pigs grow more efficiently and utilize nutrients better. Our results showed that crumble diets improved feed efficiency and nutrient digestibility in growing pigs, while diets containing extruded corn and PKM enhanced weight gain and nitrogen utilization in finishing pigs without affecting carcass quality. These findings provide practical knowledge on feeding strategies to improve productivity and profitability in swine production.

## 1. Introduction

Feed plays an important role in livestock production, representing 60–70% of the total production expenses [1]. The primary role of feedstuffs is to provide nutrients that can be efficiently digested and utilized to support maintenance and productive functions. However, the range of feed ingredients used in modern swine diets is continually evolving due to fluctuations in ingredient availability and market prices [2]. As a consequence, evaluation of ingredient quality, together with appropriate diet formulation and feed processing has become an essential tool in precision feeding, enabling more efficient nutrient utilization and improved animal performance [3].

Among the various strategies used to optimize swine nutrition, feed processing through grinding (mash or crumble), pelleting [4], and extrusion [5] has received considerable attention because these methods can improve feed efficiency, enhance nutrient availability, and promote overall growth performance. Mash diets are cost-effective but often lead to greater feed wastage and poorer feed conversion, whereas crumble diets can improve feed intake, reduce wastage, and enhance feed conversion efficiency despite higher processing costs [6]. Extrusion has also gained considerable attention due to its ability to improve nutrient digestibility, deactivate antinutritional factors, and enhance palatability through the combined effects of high temperature, pressure, and shear forces [7,8,9,10]. Previous studies have demonstrated improvements in feed efficiency with pelleting [11] and increased apparent total tract digestibility in weaned pigs with extruded diets [12].

Along with feed processing, the use of agro-industrial by-products such as palm kernel meal (PKM) has gained interest due to its cost-effectiveness and its moderate protein content and fermentable fiber fraction [13]. PKM contains structurally complex fibers that can support microbial fermentation and gut health; however, its higher fiber content may reduce nutrient digestibility when provided in mash form, largely due to particle inconsistency and reduced intake [14,15], whereas feeding PKM in crumble form may counteract these limitations, as it improves ingredient uniformity and enhances nutrient accessibility [16]. The structural refinement of crumbled feed may also facilitate more effective microbial fermentation in PKM-derived fiber, improving nutrient utilization [17]. To address this, two complementary experiments were conducted. In Experiment 1, the effects of mash and crumble diets containing 0 or 5% PKM on growth performance and nutrient digestibility were evaluated in growing pigs. In Experiment 2, finishing pigs were fed diets containing either non-extruded or extruded corn with 0 or 5% PKM to evaluate the effects of corn processing and PKM inclusion on growth performance, nutrient digestibility, and carcass characteristics. Collectively, these experiments were designed to investigate the independent and interactive effects of feed-processing methods and dietary PKM inclusion across the growing and finishing phases, thereby providing practical information for developing cost-effective and nutritionally efficient feeding programs for pigs.

## 2. Materials and Methods

### 2.1. Ethics Approval

The research protocol and procedures utilized in this study went through ethical review and received approval from (DK-2-2321_2 and_3) the Institutional Animal Use and Care Committee (IAUAC) of Dankook University in South Korea.

### 2.2. Animals, Experimental Design, Diets and Dietary Schedule

A total of 440 crossbred (Yorkshire × Landrace × Duroc) pigs were used in two independent experiments: Exp. 1 (growing pigs, *n* = 220, 63 days old) and Exp. 2 (finishing pigs, *n* = 220, 105 days old). The initial mean body weight (BW) of the growing and finishing pigs was 25.06 ± 1.82 kg and 54.80 ± 3.66 kg, respectively. In both experiments, pigs were assigned to four dietary treatments arranged in a 2 × 2 factorial design. In Exp. 1., growing pigs were fed with mash and/or crumble diets with or without 5% PKM for 6 weeks. Herein, corn, soybean meal (SBM), and PKM were all processed into both mash and crumble forms. In Exp. 2., finishing pigs were fed with mash (non-extruded) and crumble (extruded) diets with and without 5% PKM for 10 weeks. Each experiment consisted of 11 replicate pens/treatment, with five pigs (2 gilts and 3 barrows)/pen. The basal diets for both the growing (Table 1) and finishing phases (Phase 1: weeks 0–5; Phase 2: weeks 5–10) (Table 2 and Table 3) were formulated to meet or exceed the nutrient requirements recommended by the National Research Council (NRC) [18].

### 2.3. Housing and Husbandry Management

Both experiments were conducted in a fully enclosed, climate-controlled barn equipped with mechanical ventilation. Pens were furnished with adjustable front gates and positioned over fully slatted concrete floors with a 1.20-m deep manure pit beneath. Each pen was equipped with a nipple waterer and a dry self-feeder providing two eating spaces, with each feeding space measuring 35.6 cm in length and 27.9 cm in horizontal depth. Throughout the experimental period, the room temperature was maintained at 24 °C. The feeder settings were checked daily to ensure appropriate feed flow. All pigs had unrestricted (ad libitum) access to feed and water during both experiments.

### 2.4. Sampling and Clinical Analysis

Exp. 1. BW of growing pigs was recorded individually at the beginning of the experiment and at the end of weeks 3 and 6. Feed allotment and feed disappearance were measured on a pen basis at the same time points to calculate average daily gain (ADG), average daily feed intake (ADFI), and gain-to-feed ratio (G:F). To determine their apparent total tract digestibility (ATTD), pigs were offered diets containing 0.3% chromium oxide (Cr_2_O_3_) as an indigestible marker beginning in the final week of the experiment (end of week 5). At week 6, fresh fecal samples were collected from two pigs per pen, homogenized, transported to the laboratory on ice, and stored at −20 °C until analysis. Samples were dried in a convection oven at 105 °C, ground, and passed through a 1.2-mm sieve. The ATTD of dry matter (DM), nitrogen (N), and energy (E) in feed and feces was determined according to the procedures described in our previously published method [19]. Exp. 2. For finishing pigs, BW was measured at the start of the experiment and at the end of weeks 5 and 10. Growth performance indices (ADG, ADFI, and G:F) were calculated for week 0, week 5, week 10, and the overall experimental period. ATTD was determined at the end of week 10 following the same procedures used in Exp. 1. At week 10, backfat thickness (BFT, mm) was assessed at the last rib using a Piglog 105 ultrasound device (Frontmatec, Kolding, Denmark). After slaughter, the carcass weight and carcass grade were evaluated. Carcass quality (kg) was classified into grades 1+, 1, or 2 based on the Korean Institute for Animal Products Quality Evaluation (KAPE) grading system [20].

### 2.5. Statistical Analysis

Experimental data were analyzed in a completely randomized block design with pen as an experimental unit. Pigs were blocked by their initial BW. In both experiments, treatments were arranged in 2 × 2 factorial design. Analysis of variance was performed using General Linear Model (GLM) procedure of SAS (version 9.4, SAS Institute Inc., Cary, NC, USA) to evaluate the main effects of feed form, PKM inclusion, and their interaction. Statistical significance was declared at *p* < 0.05. A post-hoc power analysis was conducted using the pen-level data (α = 0.05) and the residual variance from the corresponding factorial model.

## 3. Results

Exp. 1. The effects of different forms of feed with and without PKM on growth performance and nutrient digestibility of growing pigs are summarized in Table 4. At the end of week 6, no significant (*p* > 0.05) effects of feed form, PKM inclusion, or their interaction were detected for growth performance or nutrient digestibility (*p* > 0.05). Similarly, throughout the experimental period, pigs fed mash or crumble diets containing 5% PKM did not differ significantly in growth performance from those fed the corresponding diets without PKM (*p* > 0.05). No significant feed form × PKM interaction was observed for any growth performance parameter (*p* > 0.05).

Exp. 2. The effects of feed form and PKM inclusion on the growth performance of finishing pigs are presented in Table 5. Throughout the experimental period, no significant effects of feed form, PKM inclusion, or their interaction were detected for growth-performance parameters (*p* > 0.05). Similarly, pigs fed mash (non-extruded corn) or crumble (extruded corn) diets containing 5% PKM did not differ significantly in growth performance from those fed the corresponding diets without PKM (*p* > 0.05). Also, no significant feed form × PKM interaction was observed for any growth-performance parameter (*p* > 0.05).

The effects of feed form and PKM inclusion on nutrient digestibility in finishing pigs are presented in Table 6. At the end of week 10, no significant effects of feed form, PKM inclusion, or their interaction were detected on the measured nutrient digestibility parameters (*p* > 0.05). Also, no significant feed form × PKM interaction was observed for any nutrient digestibility parameter (*p* > 0.05).

The effects of feed form and PKM inclusion on carcass characteristics and carcass grade quality in finishing pigs are presented in Table 7. At the end of week 10, no significant effects of feed form, PKM inclusion, or their interaction were detected for the measured carcass characteristics or carcass grade quality parameters (*p* > 0.05). Also, no significant feed form–PKM interaction was observed for the evaluated carcass parameters (*p* > 0.05).

## 4. Discussion

Feed form plays a vital role in determining feed utilization, although it is believed that a crumble diet has a positive effect on the performance of the host [21,22], yet several studies have indicated no difference between crumble and mash diets. For instance, Sampath et al. [19] found no impact on performance of growing-finishing pigs with mash- and/or crumble-form feed, which aligns with the present study. However, Wondra et al. [6] noted decreased ADFI in pigs fed mash diets with finer corn particle sizes (ranging from 800 to 400 μm), suggesting that smaller particle sizes in mash diets may reduce feed consumption. The contradictory findings across studies highlight that both form and particle size play crucial roles in determining feed intake and growth performance in pigs. In 2024, Jang et al. [23] reported that the inclusion of PKM in the diets of growing–finishing pigs had significantly enhanced their ADG, ADFI, and G:F ratio. However, this result was not agreed with in the present study, where pigs fed a diet containing PKM and extruded corn showed no significant changes in their performance. This discrepancy highlights the complexity and variability of dietary effects on growth, suggesting that the impact of PKM may be influenced by the feed-processing methods and/or age of pigs. Previously, Owsley et al. [24] found improved apparent digestibility of DM, starch, N, and E in growing pigs fed sorghum with particle sizes ranging from 1262 μm to 471 μm, measured at the terminal ileum, and this result was not agreed with in the current research, where no significant improvement in nutrient digestibility was observed, potentially explaining the lack of effect on the growth parameters. A meta-analysis by Jang et al. [23] addressed that many researchers used synthetic amino acids (AA) to supplement lower AA in the palm meal to meet the nutrient requirements for growing pigs, which might indirectly affect their growth. From this point, we speculated that the absence of growth performance may be closely attributed to the lack of enhanced nutrient digestibility. Yet, the precise mechanisms behind the lack of response remain unclear and require further investigation to explore the interplay between feed form, feed ingredient composition, and nutrient digestibility to better understand their collective impact on the growth performance of pigs.

Finishing pigs fed a diet containing processed corn with PKM also exhibited no improvement in their growth performance, and these findings agree with Lawlor [25], who found a similar effect on the growth performance in piglets fed an extrusion diet. However, Hongtrakul et al. [26] and Fetuga et al. [27] who reported enhanced growth performance in weaning pigs fed a diet with extrusion processing of carbohydrate sources. Several reasons can be interpreted for the conflicting results. First, it may be due to differences in corn quality, which can be influenced by extrusion parameters such as steam flow, temperature, and pressure [28]. Second, the addition of PKM with a processed corn diet. PKM is valued for its protein content and amino acid profile, but its use in pig diets is limited by the presence of anti-nutritional factors such as phytic acid, tannic acid, oxalate contents and phytin phosphorus [29]. We anticipate that these compounds might reduce feed intake and impair nutrient absorption, thereby influencing carcass composition. Earlier studies have consistently shown that reducing particle size enhances nutrient digestibility. For instance, Li et al. [30] reported that a diet containing extruded corn increased the apparent digestibility of DM, N, and energy in piglets. Similarly, Liu et al. [31] suggested that the extrusion process of corn and broken rice had increased the ATTD of DM and E in weaning pigs. However, in this study, pigs fed a diet containing processed corn showed no significant improvement in digestibility; we supposed that these findings could be due to the increased enzyme susceptibility and reduced starch resistant content of the diet [32]. Apart from this, several mechanisms can be interpreted for the lack of nutrient digestibility parameters in finishing pigs. First, if the extrusion process did not achieve an adequate level of starch gelatinization in the corn, the digestibility of DM and E would not be improved [33]. Second, pigs have limited ability to break down fiber, particularly in the small intestine [34]. Although extrusion has enhanced the digestibility of starch from corn, we proposed that the presence of fiber in PKM [35] might restrict the overall digestibility and thus show no improvement in digestibility. BFT serves as an important indicator of carcass composition in pork production [36]. Indeed, carcass quality is typically assessed for palatability. The inclusion of alternative protein sources such as PKM in swine diets has gained popularity due to nutritional benefits. However, their effects on carcass quality in finishing pigs remain a subject of ongoing investigation, with mixed findings reported in the literature. For instance, Potter et al. [37] reported that pigs fed a pellet diet had increased carcass yield and BFT compared to those fed a mash diet. However, in this study, there was no significant difference in BFT or carcass grade quality in finishing pigs, which aligns with Pettersson and Björklund [38], who found similar outcomes. To date, no research has compared the efficacy of these feed forms in combination with PKM on carcass quality in finishing pigs; thus, a sufficient comparison could not be made, and this needs further investigation. Overall, the findings indicate that no statistically significant differences were detected among the evaluated feed-form and PKM treatments for the measured growth performance, nutrient digestibility, and carcass characteristics. Yet, the limited statistical power (Supplementary Figures) of the experimental design restricts the ability to exclude relatively small treatment effects and thus the present results are considered as evidence of the absence of detected treatment differences under the conditions of this experiment rather than evidence of treatment equivalence.

## 5. Conclusions

The present study had insufficient (less than 80%) statistical power to show possible differences between the treatment groups, suggesting that current findings are preliminary evidence regarding the use of PKM and different feed-processing strategies in pig diets; however, further studies with greater experimental replication are warranted to validate these findings and clarify potential treatment effects.

## Figures and Tables

**Table 1:** Composition of growing pig diets (as-fed basis).

Items	−PKM		+PKM	
Ingredients (%)	Mash	Crumble	Mash	Crumble
Corn	74.97	75.47	70.97	71.42
Soybean meal	19.16	18.1	18.16	17.15
Palm kernel meal	-	-	5.00	5.00
Tallow	0.62	1.18	0.62	1.18
Molasses	2.00	2.00	2.00	2.00
MDCP	1.04	1.01	1.04	1.01
Limestone	0.82	0.82	0.82	0.82
Lysine (78%)	0.46	0.48	0.46	0.48
Methionine (98%)	0.06	0.06	0.06	0.06
Threonine (98%)	0.15	0.16	0.15	0.16
Tryptophan (98%)	0.02	0.02	0.02	0.02
Salt	0.20	0.20	0.20	0.20
Mineral mix ^1^	0.20	0.20	0.20	0.20
Vitamin mix ^2^	0.20	0.20	0.20	0.20
Choline (25%)	0.10	0.10	0.10	0.10
Total	100.00	100.00	100.00	100.00
Calculated value				
Crude protein, %	16.00	16.00	16.00	16.00
Metabolizable energy, kcal/kg	3300	3300	3300	3300
Calcium, %	0.66	0.66	0.66	0.66
Phosphorus, %	0.56	0.56	0.56	0.56
Lysine, %	1.12	1.12	1.12	1.12
Methionine, %	0.32	0.32	0.32	0.32
Threonine, %	0.72	0.72	0.72	0.72
Tryptophan, %	0.19	0.19	0.19	0.19
Crude Fat, %	3.52	4.04	3.52	4.04
Crude Fiber, %	2.23	3.04	2.23	3.04
Crude Ash, %	4.13	4.19	4.13	4.19

^1^ Provided per kg diet: Fe, 100 mg as ferrous sulfate; Cu, 17 mg as copper sulfate; Mn, 17 mg as manganese oxide; I, 0.5 mg as potassium iodide; and Se, 0.3 mg as sodium selenite. ^2^ Provided per kilogram of diet: vitamin A, 10,800 IU; vitamin D3, 4000 IU; vitamin E, 40 IU; vitamin K3, 4 mg; vitamin B1, 6 mg; vitamin B2, 12 mg; vitamin B6, 6 mg; vitamin B12, 0.05 mg; biotin, 0.2 mg; folic acid, 2 mg; niacin, 50 mg; D-calcium pantothenate, 25 mg.

**Table 2:** Composition of finishing pigs (as-fed basis).

Items	Phase 1 (Week 0–5)			
	−PKM		+PKM	
Ingredients (%)	Mash	Crumble	Mash	Crumble
Corn	77.83	67.34	73.31	62.84
EP corn	-	10.00	-	10.00
Soybean meal	16.90	17.21	16.47	16.78
Palm kernel meal	-	-	5.00	5.00
Tallow	0.43	0.60	0.49	0.65
Molasses	2.00	2.00	2.00	2.00
MDCP	0.89	0.91	0.74	0.75
Limestone	0.75	0.75	0.76	0.76
Lysine (78%)	0.35	0.34	0.40	0.39
Methionine (98%)	0.03	0.03	0.01	0.01
Threonine (98%)	0.11	0.11	0.10	0.10
Tryptophan (98%)	0.01	0.01	0.02	0.02
Salt	0.20	0.20	0.20	0.20
Mineral mix ^1^	0.20	0.20	0.20	0.20
Vitamin mix ^2^	0.20	0.20	0.20	0.20
Choline (25%)	0.10	0.10	0.10	0.10
Total	100.00	100.00	100.00	100.00
Calculated value				
Crude protein, %	15.00	15.00	15.00	15.00
Metabolizable energy, kcal/kg	3300	3300	3300	3300
Calcium, %	0.59	0.59	0.59	0.59
Phosphorus, %	0.52	0.52	0.52	0.52
Lysine, %	0.97	0.97	0.97	0.97
Methionine, %	0.28	0.28	0.28	0.28
Threonine, %	0.64	0.64	0.64	0.64
Tryptophan, %	0.17	0.17	0.17	0.17
Crude Fat, %	3.39	3.42	4.25	4.26
Crude Fiber, %	2.20	2.19	2.92	2.92
Crude Ash, %	3.81	3.83	3.80	3.82

^1^ Provided per kg diet: Fe, 100 mg as ferrous sulfate; Cu, 17 mg as copper sulfate; Mn, 17 mg as manganese oxide; I, 0.5 mg as potassium iodide; and Se, 0.3 mg as sodium selenite. ^2^ Provided per kilogram of diet: vitamin A, 10,800 IU; vitamin D3, 4000 IU; vitamin E, 40 IU; vitamin K3, 4 mg; vitamin B1, 6 mg; vitamin B2, 12 mg; vitamin B6, 6 mg; vitamin B12, 0.05 mg; biotin, 0.2 mg; folic acid, 2 mg; niacin, 50 mg; D-calcium pantothenate, 25 mg.

**Table 3:** Composition of finishing pig (as-fed basis).

Item	Phase 2 (Week 5–10)			
	−PKM		+PKM	
Ingredients (%)	Mash	Crumble	Mash	Crumble
Corn	83.49	73.01	77.91	68.45
EP corn	-	10.00	-	10.00
Soybean meal	11.69	12.01	11.78	11.10
Palm kernel meal	-	-	5.00	5.00
Tallow	0.16	0.32	0.81	0.96
Molasses	2.00	2.00	2.00	2.00
MDCP	0.78	0.78	0.61	0.61
Limestone	0.67	0.68	0.66	0.66
Lysine (78%)	0.36	0.35	0.39	0.38
Methionine (98%)	0.02	0.02	0.01	0.01
Threonine (98%)	0.11	0.11	0.10	0.10
Tryptophan (98%)	0.02	0.02	0.03	0.03
Salt	0.20	0.20	0.20	0.20
Mineral mix ^1^	0.20	0.20	0.20	0.20
Vitamin mix ^2^	0.20	0.20	0.20	0.20
Choline (25%)	0.10	0.10	0.10	0.10
Total	100.00	100.00	100.00	100.00
Calculated value				
Crude protein, %	13.00	13.00	13.00	13.00
Metabolizable energy, kcal/kg	3300	3300	3300	3300
Calcium, %	0.52	0.52	0.52	0.52
Phosphorus, %	0.47	0.47	0.47	0.47
Lysine, %	0.84	0.84	0.84	0.84
Methionine, %	0.25	0.25	0.25	0.25
Threonine, %	0.56	0.56	0.56	0.56
Tryptophan, %	0.15	0.15	0.15	0.15
Crude Fat, %	3.24	3.26	4.33	4.34
Crude Fiber, %	2.11	2.10	3.34	3.34
Crude Ash, %	3.39	3.40	3.43	3.43

^1^ Provided per kg diet: Fe, 100 mg as ferrous sulfate; Cu, 17 mg as copper sulfate; Mn, 17 mg as manganese oxide; I, 0.5 mg as potassium iodide; and Se, 0.3 mg as sodium selenite. ^2^ Provided per kilogram of diet: vitamin A, 10,800 IU; vitamin D3, 4000 IU; vitamin E, 40 IU; vitamin K3, 4 mg; vitamin B1, 6 mg; vitamin B2, 12 mg; vitamin B6, 6 mg; vitamin B12, 0.05 mg; biotin, 0.2 mg; folic acid, 2 mg; niacin, 50 mg; D-calcium pantothenate, 25 mg.

**Table 4:** Effect of feed form modification on the growth performance and nutrient digestibility in growing pigs.

Items	+PKM		−PKM		SEM	*p*-Value		
	Mash	Crumble	Mash	Crumble		Feed Form	PKM	Interaction
Growth performance								
BW, kg								
Initial	25.06	25.07	25.06	25.08	0.005	0.995	0.997	0.992
Week 3	38.85	39.07	38.57	38.92	0.31	0.733	0.661	0.920
Week 6	54.75	55.35	54.13	54.97	0.65	0.575	0.423	0.894
ADG, g								
Week 3	657	667	643	660	15	0.516	0.395	0.835
Week 6	757	775	741	764	18	0.496	0.294	0.894
Overall	707	721	692	712	16	0.502	0.333	0.866
ADFI, g								
Week 3	1468	1473	1444	1466	28	0.616	0.661	0.766
Week 6	2029	2044	1995	2027	35	0.514	0.546	0.834
Overall	1748	1758	1719	1747	31	0.556	0.593	0.803
G:F, kg								
Week 3	2.239	2.209	2.248	2.223	0.016	0.439	0.068	0.855
Week 6	2.685	2.644	2.700	2.657	0.034	0.660	0.200	0.980
Overall	2.477	2.442	2.489	2.456	0.024	0.570	0.128	0.971
Nutrient digestibility, %								
Week 6								
DM	76.28	76.95	75.88	76.61	0.69	0.599	0.329	0.967
N	73.42	74.63	73.15	74.06	0.55	0.464	0.080	0.785
E	75.93	76.64	75.64	76.20	0.43	0.408	0.162	0.858

Abbreviation: BW, body weight; ADG, average daily gain; ADFI, average daily feed intake; G:F, gain-to-feed ratio; DM, dry matter; N, nitrogen; E, energy. Mash (corn+ soybean meal); Crumble (corn + soybean meal); −PKM (palm kernel meal, 0%); + PKM (with 5% PKM). SEM, standard error of means.

**Table 5:** Effect of feed form (corn processing) with and without a PKM diet on growth performance of finishing pigs.

Items	+PKM		−PKM			*p*-Value		
	Mash	Crumble	Mash	Crumble	SEM	Corn	PKM	Interaction
BW, kg								
Initial	54.83	54.78	54.82	54.77	1.13	0.962	0.996	0.997
Week 5	79.35	80.63	80.98	81.63	1.80	0.556	0.391	0.703
Week 10	113.21	115.14	116.13	117.32	1.91	0.420	0.190	0.847
ADG, g								
Week 5	701	739	747	757	18	0.190	0.075	0.426
Week 10	967	986	1004	1030	39	0.516	0.244	0.935
Overall	834	862	875	894	23	0.315	0.114	0.817
ADFI, g								
Week 5	2195	2236	2275	2279	43	0.604	0.162	0.673
Week 10	2725	2803	2890	2870	63	0.154	0.072	0.441
Overall	2433	2484	2539	2532	50	0.664	0.135	0.569
G:F, kg								
Week 5	3.131	3.026	3.046	3.011	0.051	0.115	0.268	0.394
Week 10	2.818	2.843	2.878	2.786	0.063	0.435	0.786	0.553
Overall	2.917	2.882	2.902	2.832	0.030	0.133	0.332	0.686

Abbreviation: BW, body weight; ADG, average daily gain; ADFI, average daily feed intake; G:F, gain-to-feed ratio; Mash (non-extruded corn); Crumble (extruded corn); −PKM (palm kernel meal, 0%); +PKM (with 5% PKM). SEM, standard error of means.

**Table 6:** Effect of feed form (corn processing) with and without PKM diet on nutrient digestibility in finishing pigs.

	+PKM		−PKM			*p*-Value		
Week 6	Mash	Crumble	Mash	Crumble	SEM	Corn	PKM	Interaction
Dry matter	73.91	75.29	74.62	75.74	0.75	0.140	0.487	0.871
Nitrogen	71.48	72.54	71.99	73.03	0.62	0.224	0.557	0.992
Energy	72.81	73.71	73.26	74.35	0.66	0.149	0.422	0.886

Abbreviation: Mash (non-extruded corn); Crumble (extruded corn); −PKM (palm kernel meal, 0%); +PKM (with 5% PKM). SEM, standard error of means.

**Table 7:** Effect of feed form (corn processing) with and without PKM diet on carcass grade quality in finishing pigs.

	+PKM		−PKM			*p*-Value		
	Mash	Crumble	Mash	Crumble	SEM	Corn	PKM	Interaction
Carcass weight, kg	89.18	89.27	89.42	89.67	0.85	0.835	0.702	0.921
Backfat thickness, mm	16.24	16.44	16.84	16.91	0.31	0.658	0.083	0.839
1+, %	20.00	23.64	25.45	27.27	-	-	-	-
1, %	38.18	40.00	43.64	45.45	-	-	-	-
2, %	41.82	36.36	30.91	27.27	-	-	-	-

Abbreviation: Mash (non-extruded corn); Crumble (extruded corn); −PKM (palm kernel meal, 0%); +PKM (with 5% PKM). SEM, standard error of means.

## Data Availability

The data presented in this study are available on request from the corresponding author due to privacy reasons.

## Supplementary Materials

The following supporting information can be downloaded at https://www.mdpi.com/article/10.3390/vetsci13090963/s1.

## Abbreviations

The following abbreviations are used in this manuscript:

PKM	Palm Kernal Meal
N	Nitrogen
CP	Crude Protein
DM	Dry Matter
E	Energy
G:F	Gain-to-Feed Ratio
ADG	Average Daily Gain
ADFI	Average Daily Feed Intake
ATTD	Apparent Total Tract Digestibility
BW	Body Weight
