# Supplementary material for: Palm Kernel Meal Inclusion Does Not Compromise Performance of Growing and Finishing Pigs Regardless of Feed Form or Corn Processing
Source: Vet Sci. 2026 Sep 14;13(9):963. doi: 10.3390/vetsci13090963 (PMC13611954; doi:10.3390/vetsci13090963)
Supplement: Supplementary file 1 [file vetsci-13-00963-s001.zip › vetsci-4421540-supplementary.pdf]

EXP. 1

A factor： mash feed or crumble feed

B factor： PKM diet

BW

WEEK3

A factor

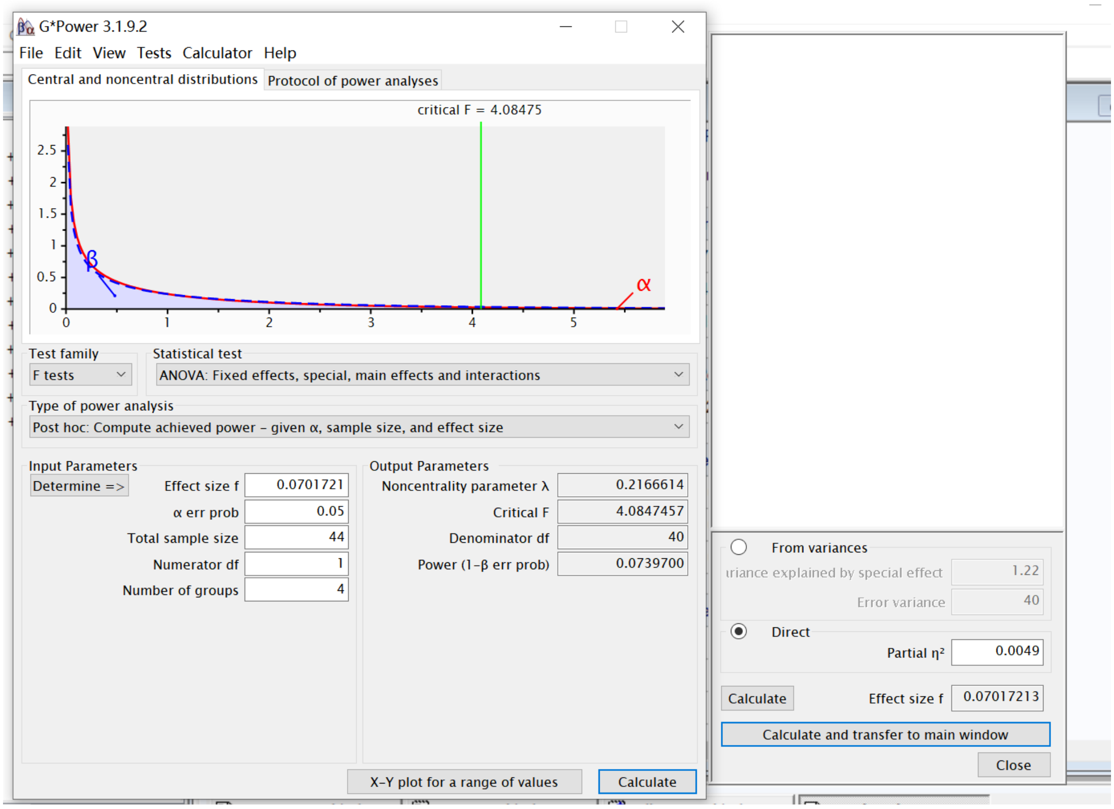

B factor

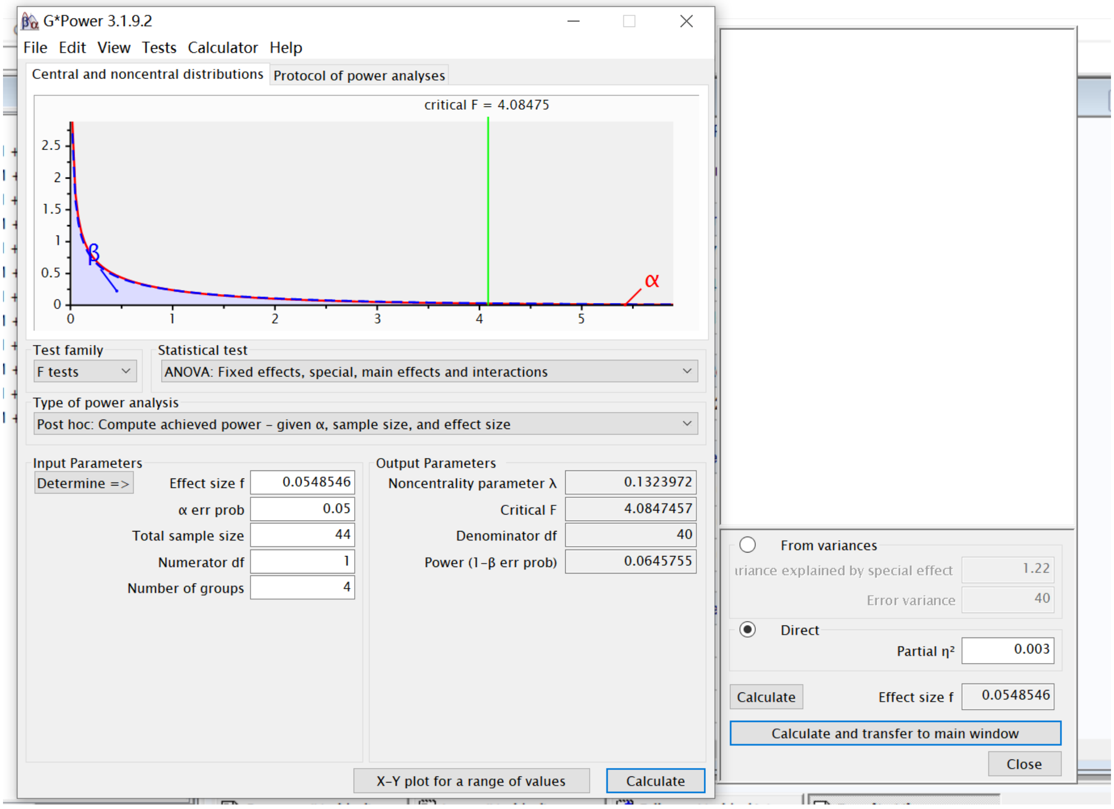
G*Power 3.1.9.2
File Edit View Tests Calculator Help
Central and noncentral distributions
Protocol of power analyses
critical F = 4.08475
Test family
F tests
Statistical test
ANOVA: Fixed effects, special, main effects and interactions
Type of power analysis
Post hoc: Compute achieved power – given α, sample size, and effect size
Input Parameters
Determine =>
Effect size f 0.0548546
α err prob 0.05
Total sample size 44
Numerator df 1
Number of groups 4
Output Parameters
Noncentrality parameter λ 0.1323972
Critical F 4.0847457
Denominator df 40
Power (1–β err prob) 0.0645755
X–Y plot for a range of values
Calculate
From variances
ıriance explained by special effect 1.22
Error variance 40
Direct
Partial η² 0.003
Calculate
Effect size f 0.0548546
Calculate and transfer to main window
Close

A ×B

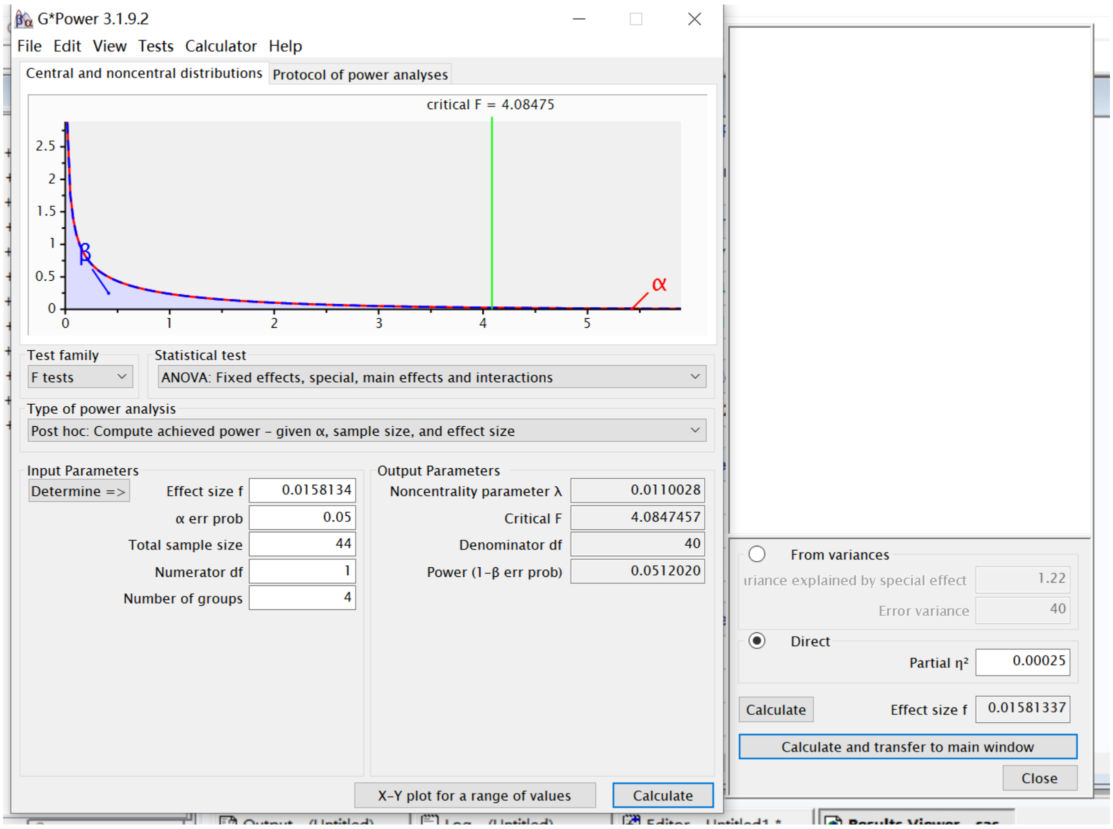
G*Power 3.1.9.2
File Edit View Tests Calculator Help
Central and noncentral distributions
Protocol of power analyses
critical F = 4.08475
Test family
F tests
Statistical test
ANOVA: Fixed effects, special, main effects and interactions
Type of power analysis
Post hoc: Compute achieved power – given α, sample size, and effect size
Input Parameters
Determine =>
Effect size f 0.0158134
α err prob 0.05
Total sample size 44
Numerator df 1
Number of groups 4
Output Parameters
Noncentrality parameter λ 0.0110028
Critical F 4.0847457
Denominator df 40
Power (1–β err prob) 0.0512020
X–Y plot for a range of values
Calculate
From variances
ıriance explained by special effect 1.22
Error variance 40
Direct
Partial η² 0.00025
Calculate
Effect size f 0.01581337
Calculate and transfer to main window
Close

WEEK6

BW

A factor

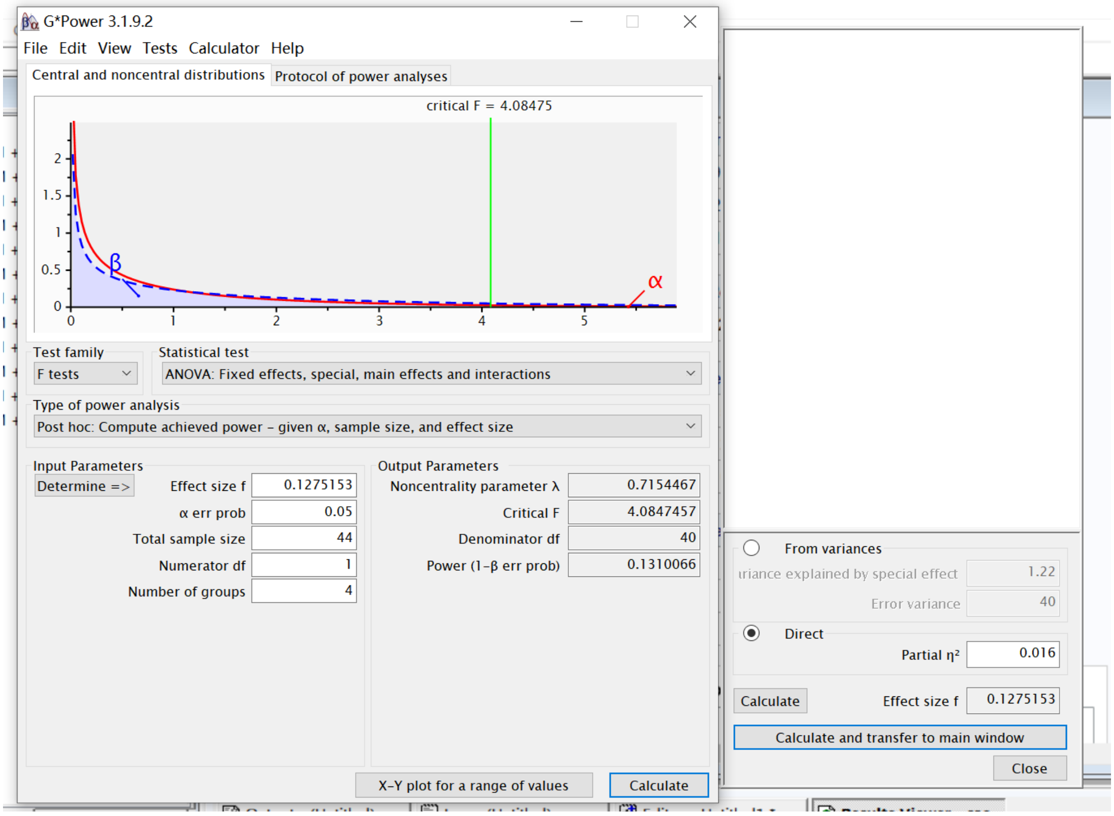

B factor

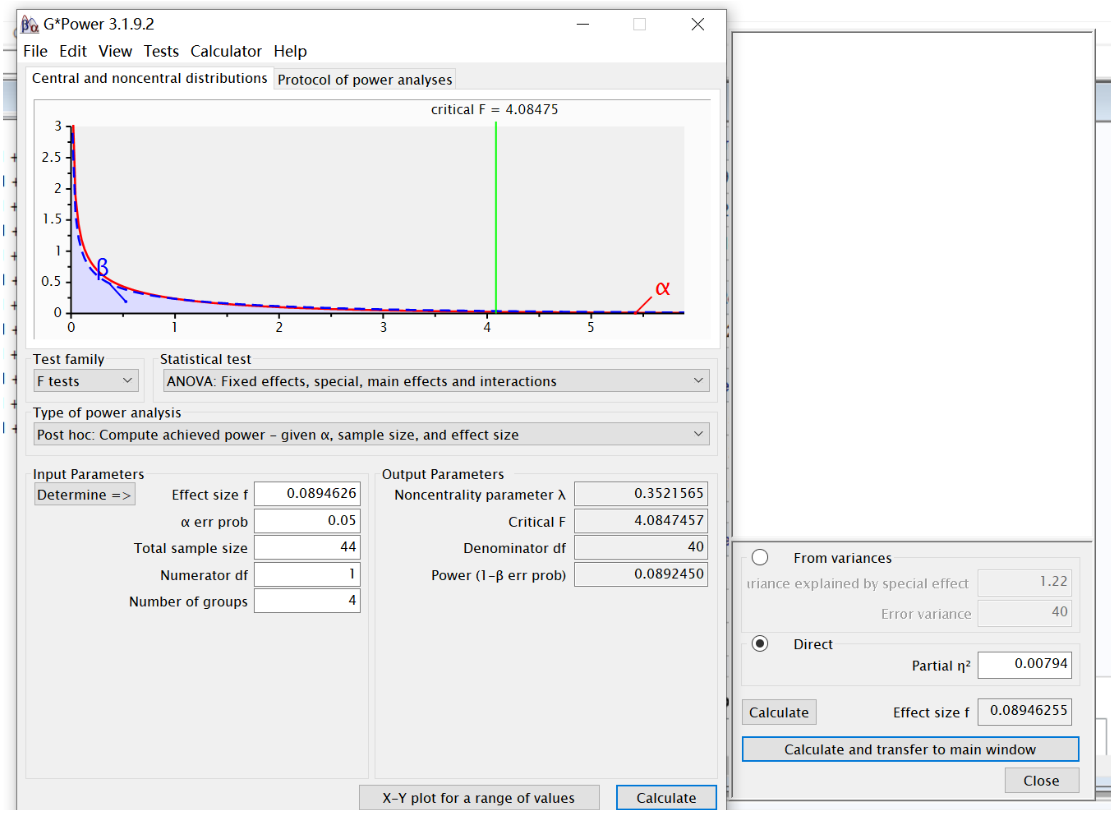

A ×B

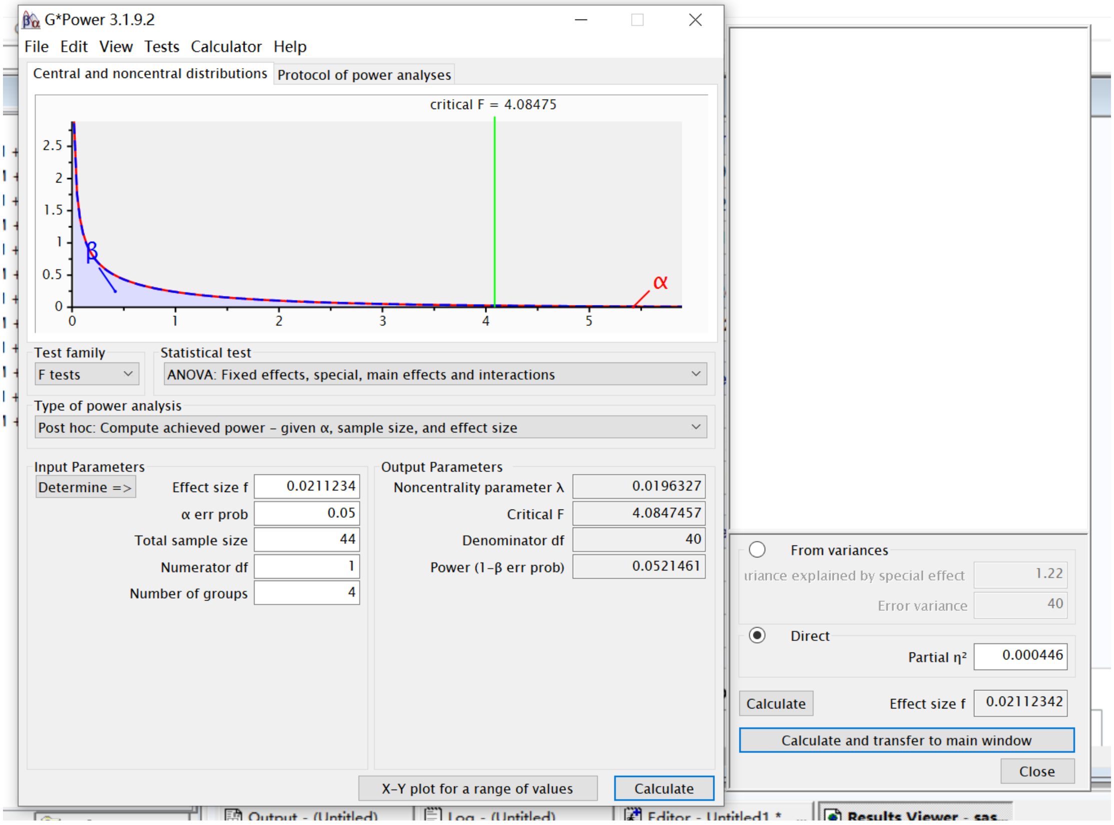

ADG overall

A factor

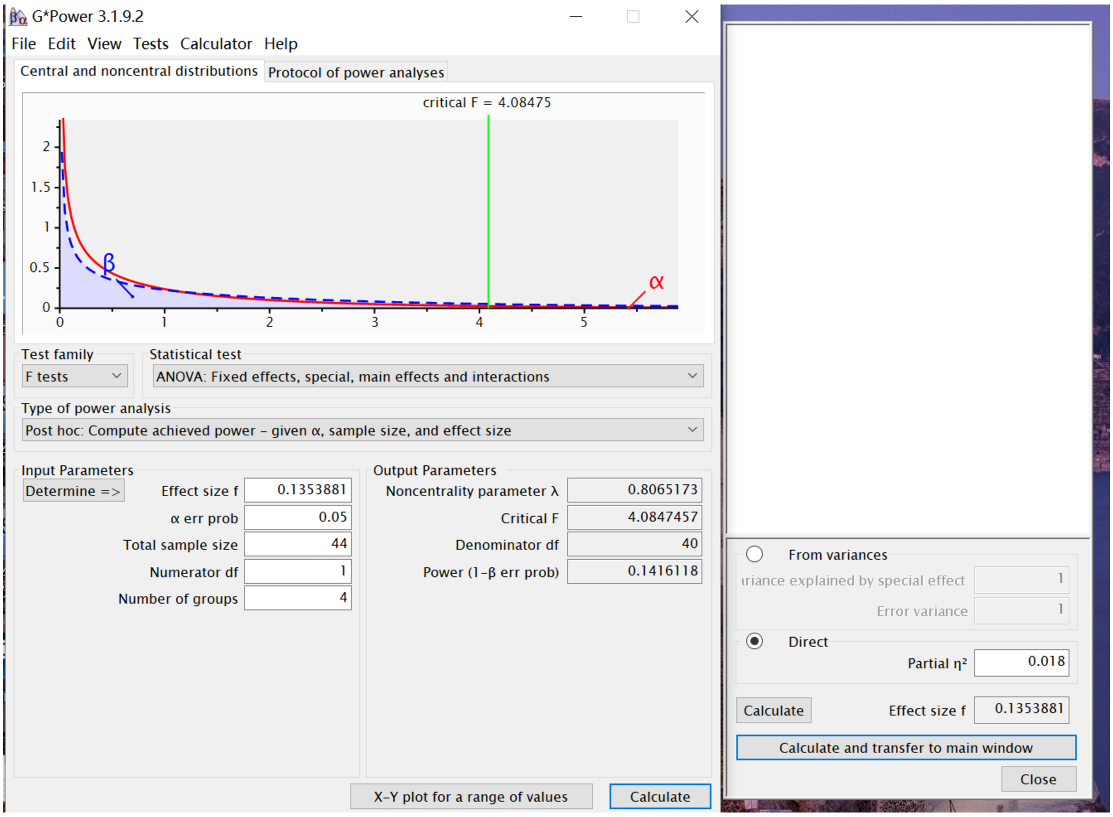

B factor

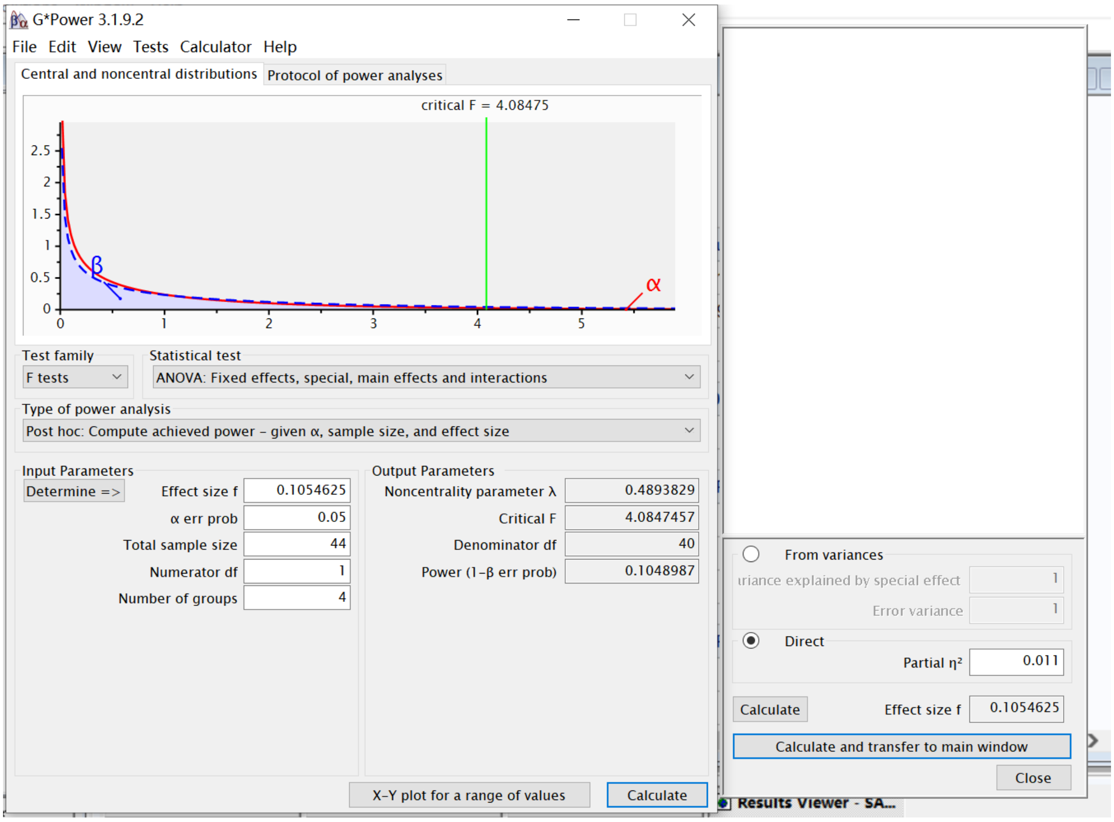

G*Power 3.1.9.2
File Edit View Tests Calculator Help
Central and noncentral distributions
Protocol of power analyses
critical F = 4.08475
Test family
F tests
Statistical test
ANOVA: Fixed effects, special, main effects and interactions
Type of power analysis
Post hoc: Compute achieved power – given α, sample size, and effect size
Input Parameters
Determine =>
Effect size f 0.1054625
α err prob 0.05
Total sample size 44
Numerator df 1
Number of groups 4
Output Parameters
Noncentrality parameter λ 0.4893829
Critical F 4.0847457
Denominator df 40
Power (1−β err prob) 0.1048987
X-Y plot for a range of values
Calculate
From variances
Error variance 1
Direct
Partial η² 0.011
Calculate
Effect size f 0.1054625
Calculate and transfer to main window
Close

A×B

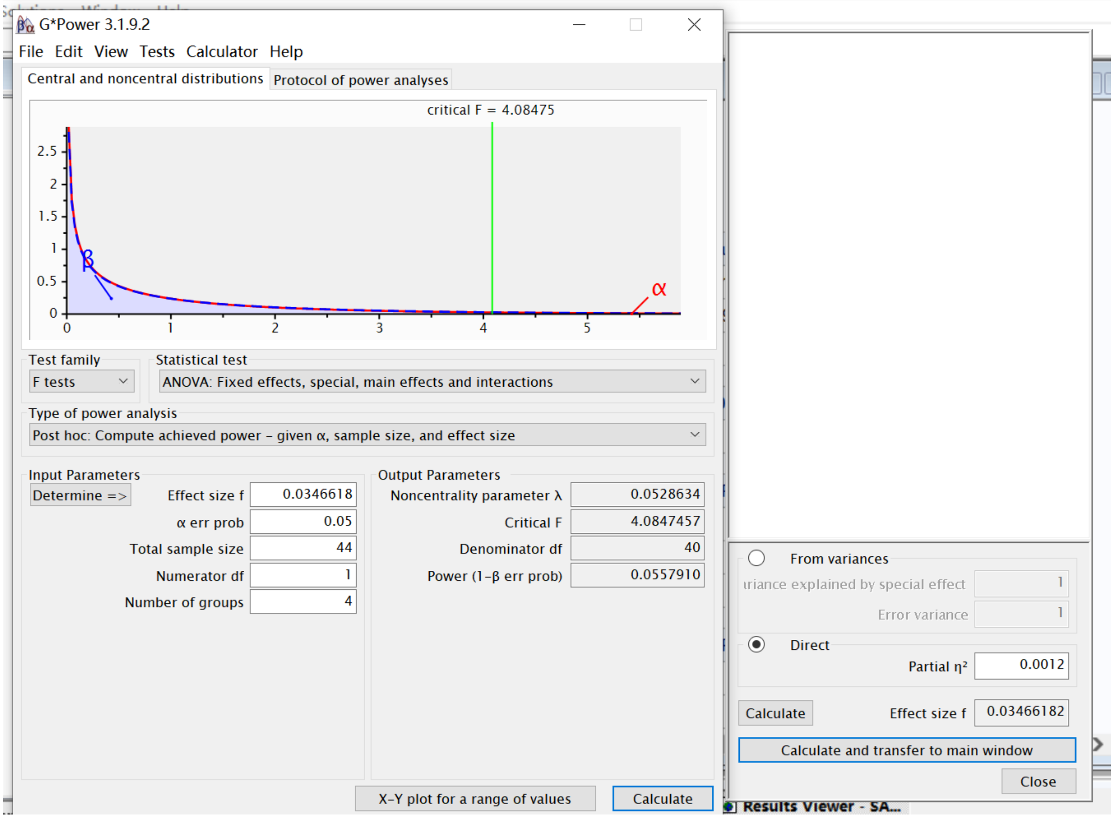

G*Power 3.1.9.2
File Edit View Tests Calculator Help
Central and noncentral distributions
Protocol of power analyses
critical F = 4.08475
Test family
F tests
Statistical test
ANOVA: Fixed effects, special, main effects and interactions
Type of power analysis
Post hoc: Compute achieved power – given α, sample size, and effect size
Input Parameters
Determine =>
Effect size f 0.0346618
α err prob 0.05
Total sample size 44
Numerator df 1
Number of groups 4
Output Parameters
Noncentrality parameter λ 0.0528634
Critical F 4.0847457
Denominator df 40
Power (1−β err prob) 0.0557910
X-Y plot for a range of values
Calculate
From variances
Error variance 1
Direct
Partial η² 0.0012
Calculate
Effect size f 0.03466182
Calculate and transfer to main window
Close

ADFI overall

A factor

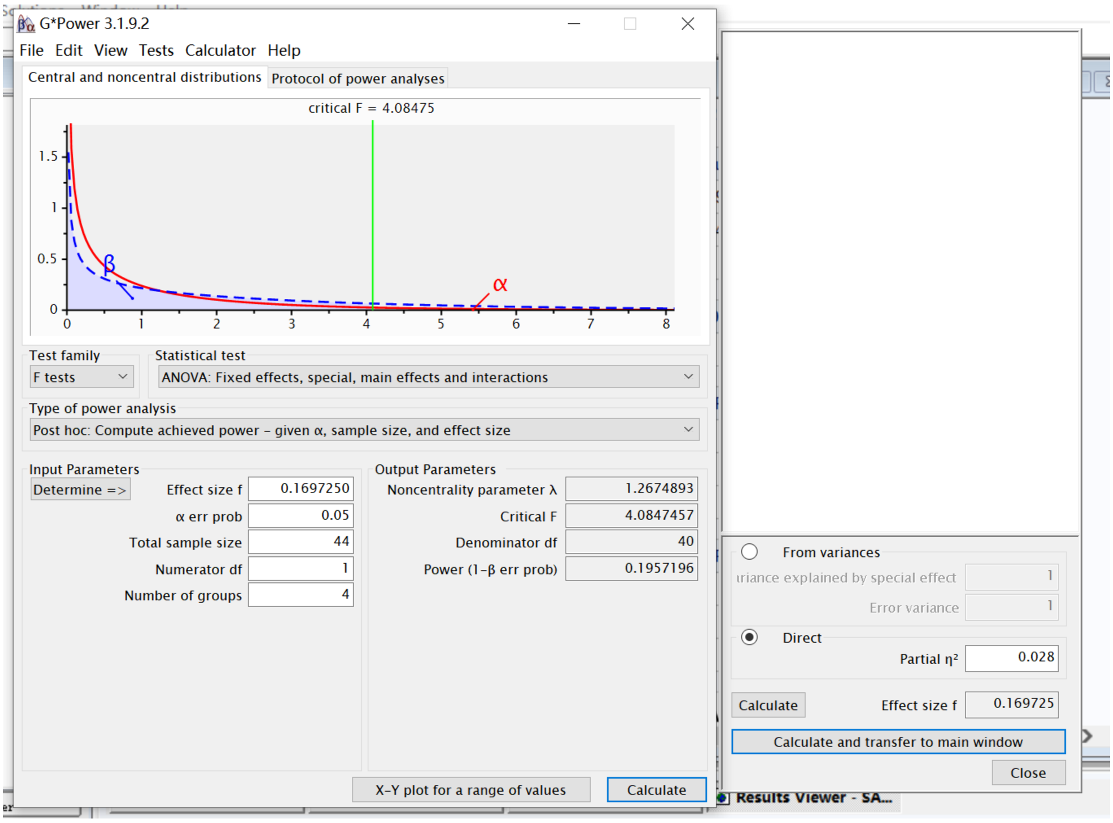

B factor

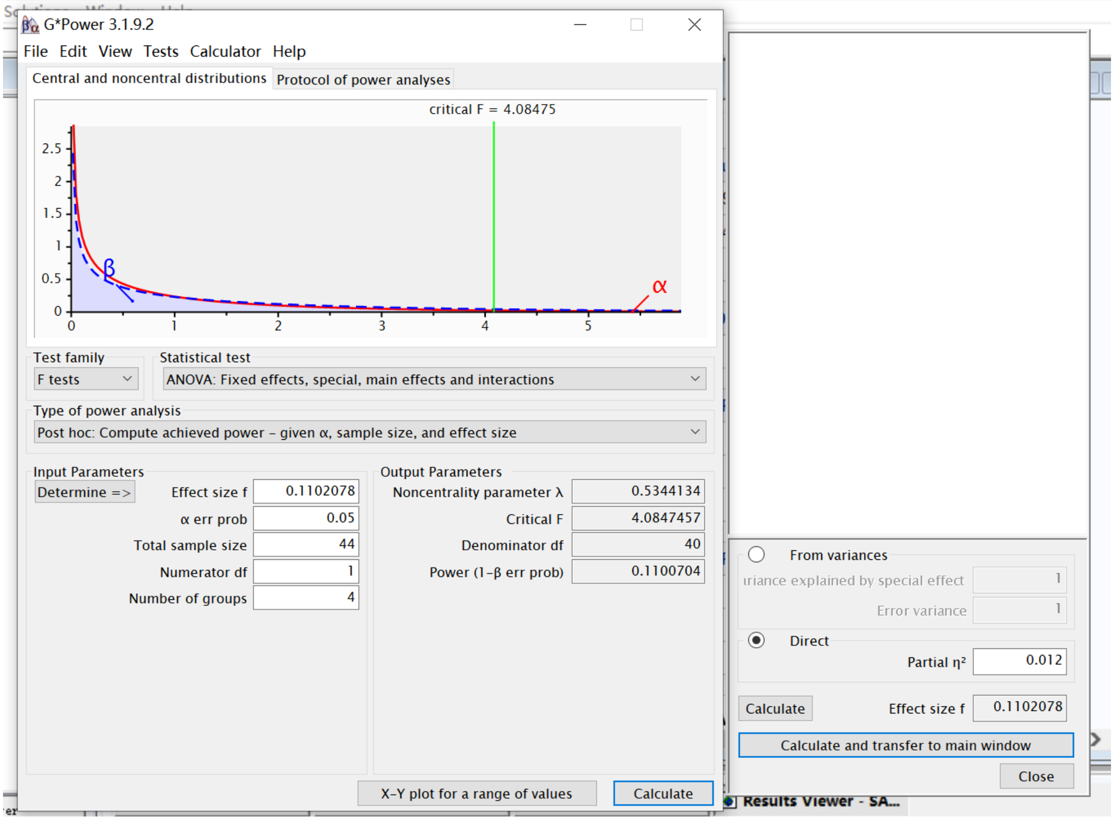

A ×B

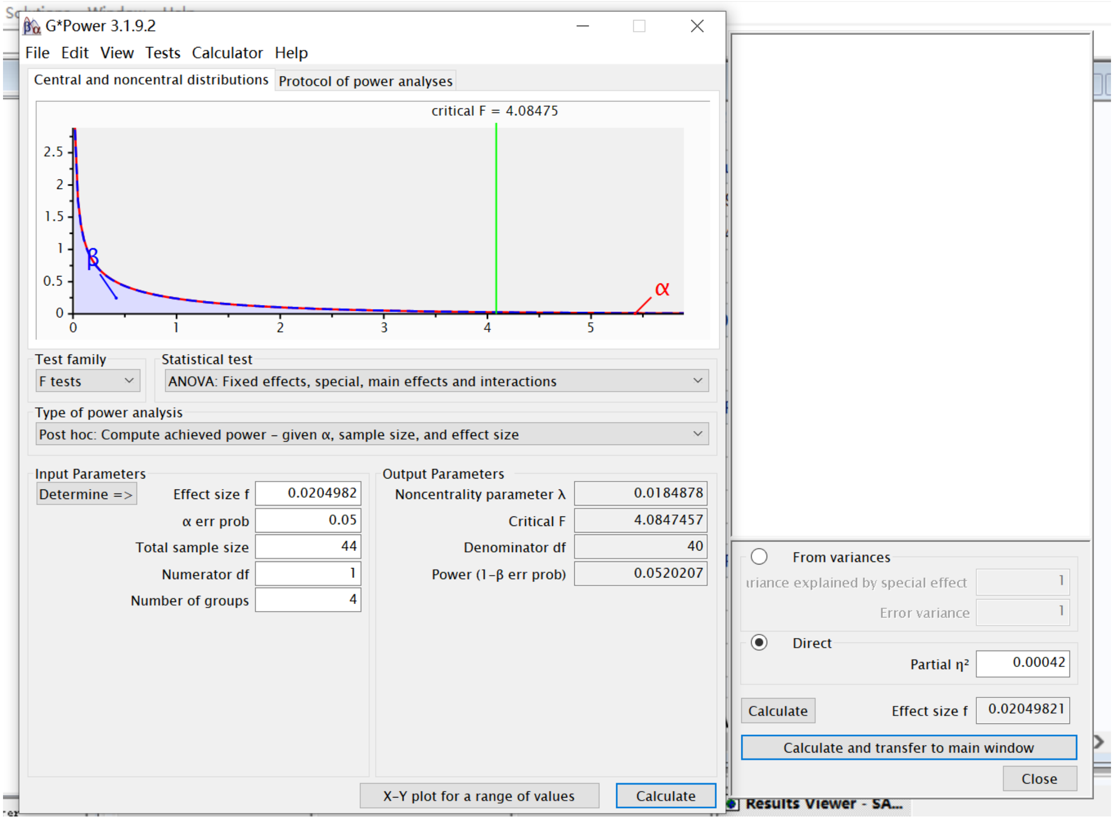

G:F overall

A factor

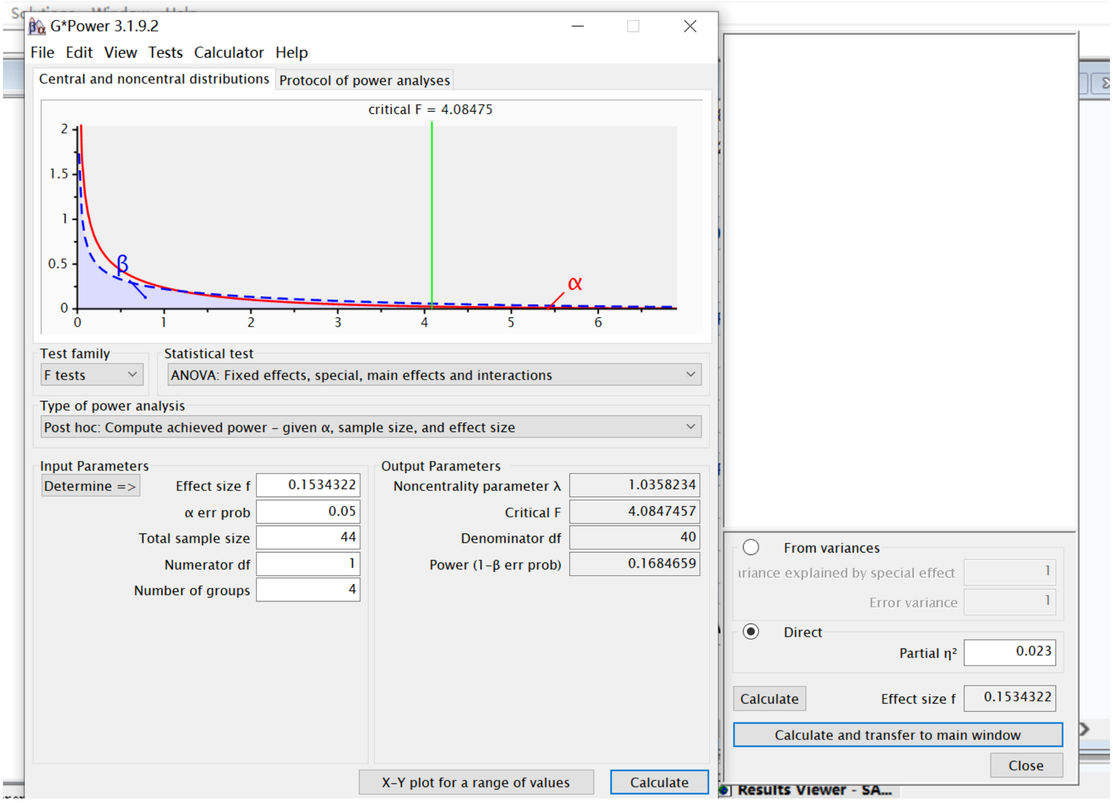

B factor

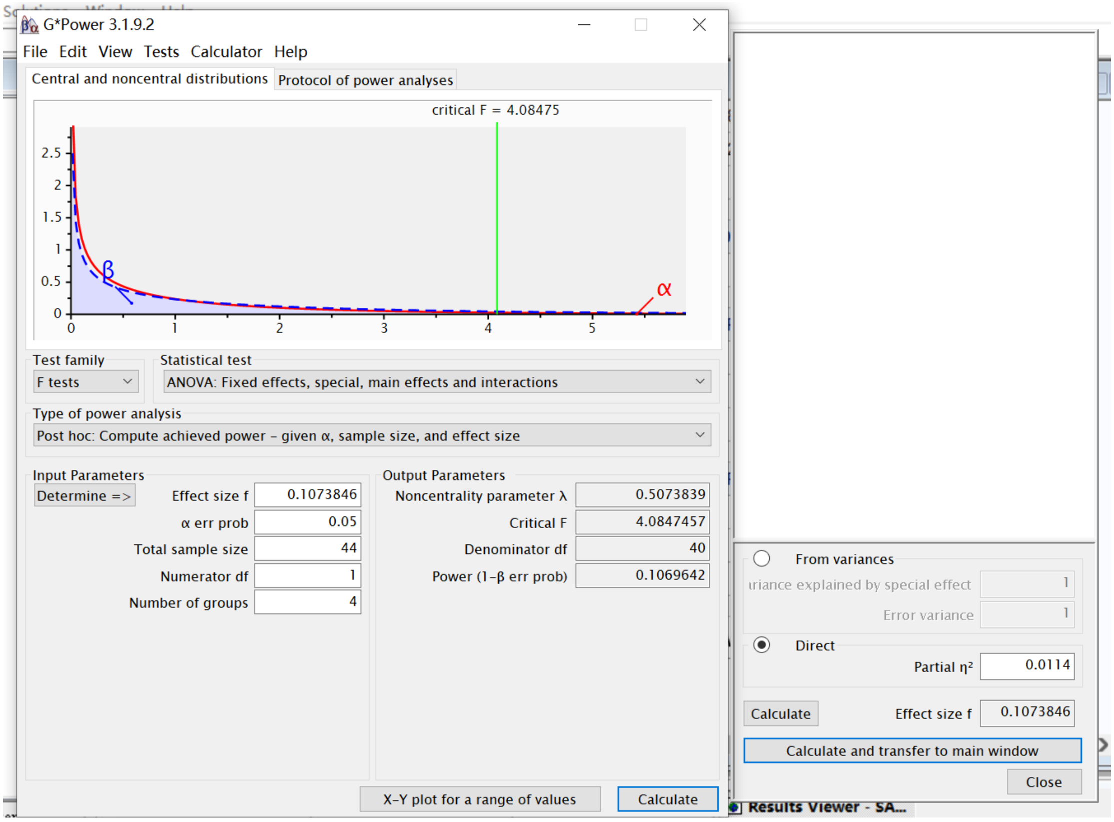

G*Power 3.1.9.2
File Edit View Tests Calculator Help
Central and noncentral distributions
Protocol of power analyses
critical F = 4.08475
Test family
F tests
Statistical test
ANOVA: Fixed effects, special, main effects and interactions
Type of power analysis
Post hoc: Compute achieved power – given α, sample size, and effect size
Input Parameters
Determine =>
Effect size f 0.1073846
α err prob 0.05
Total sample size 44
Numerator df 1
Number of groups 4
Output Parameters
Noncentrality parameter λ 0.5073839
Critical F 4.0847457
Denominator df 40
Power (1–β err prob) 0.1069642
X-Y plot for a range of values
Calculate
From variances
Error variance 1
Direct
Partial η² 0.0114
Calculate
Effect size f 0.1073846
Calculate and transfer to main window
Close

A ×B

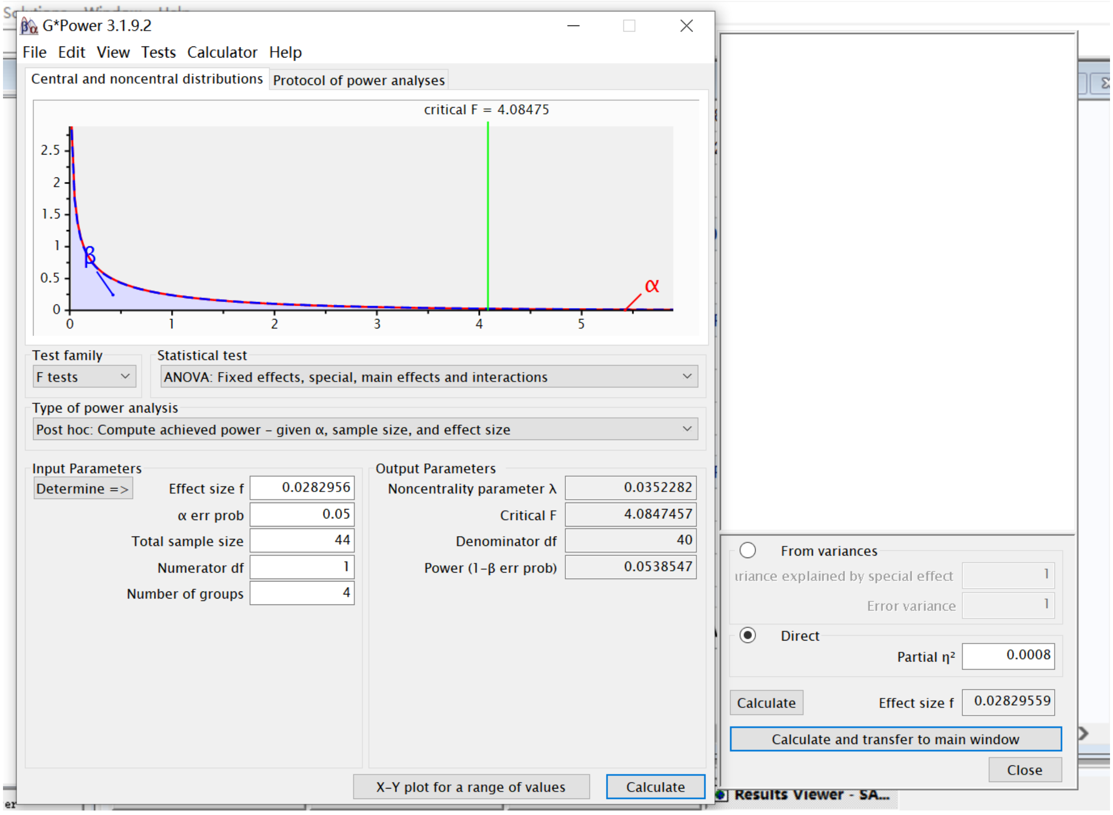

G*Power 3.1.9.2
File Edit View Tests Calculator Help
Central and noncentral distributions
Protocol of power analyses
critical F = 4.08475
Test family
F tests
Statistical test
ANOVA: Fixed effects, special, main effects and interactions
Type of power analysis
Post hoc: Compute achieved power – given α, sample size, and effect size
Input Parameters
Determine =>
Effect size f 0.0282956
α err prob 0.05
Total sample size 44
Numerator df 1
Number of groups 4
Output Parameters
Noncentrality parameter λ 0.0352282
Critical F 4.0847457
Denominator df 40
Power (1–β err prob) 0.0538547
X-Y plot for a range of values
Calculate
From variances
Error variance 1
Direct
Partial η² 0.0008
Calculate
Effect size f 0.02829559
Calculate and transfer to main window
Close

DM

A factor

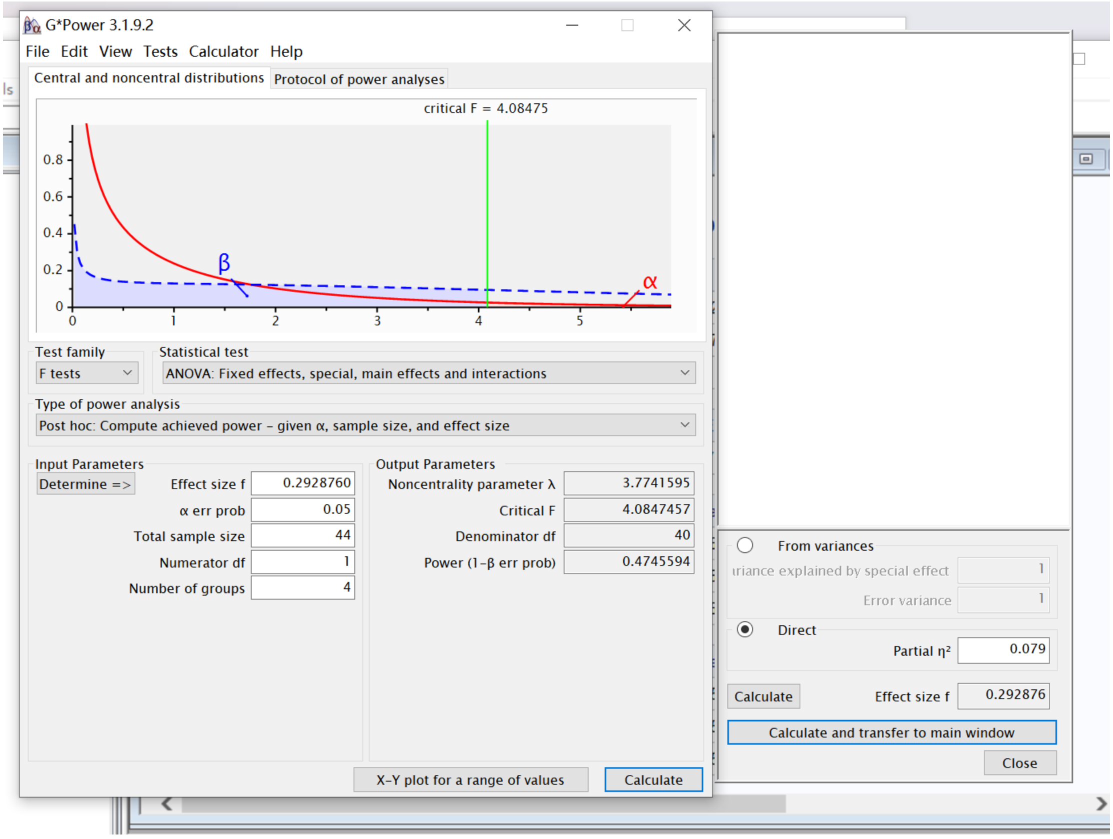

B factor

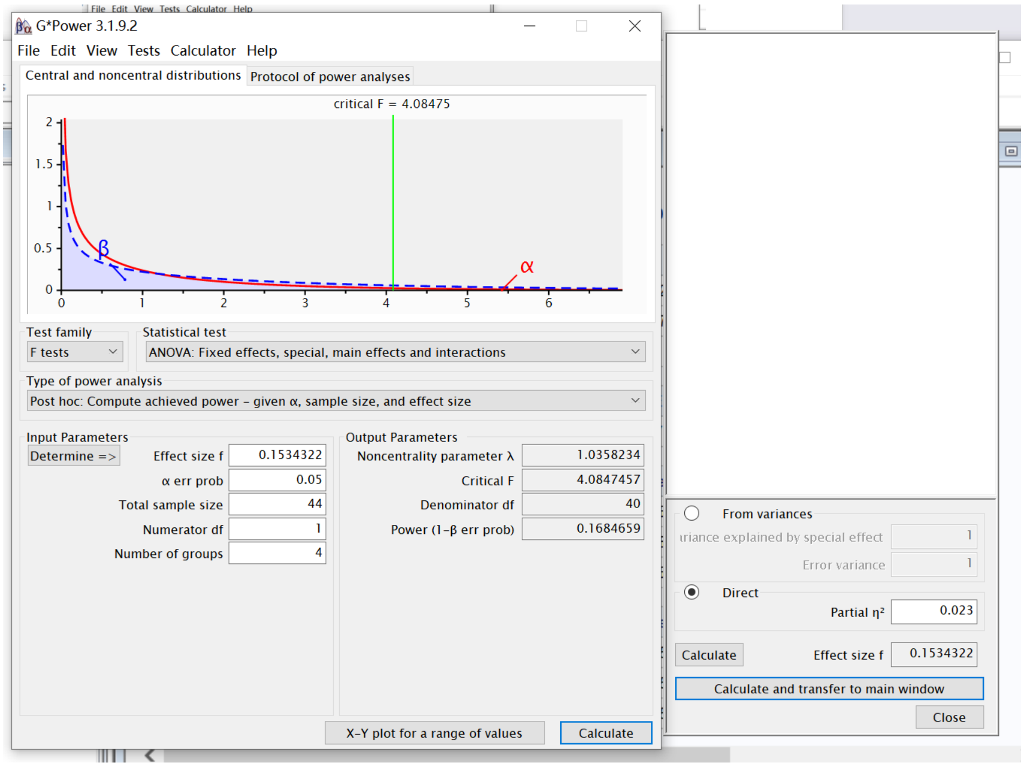

A ×B

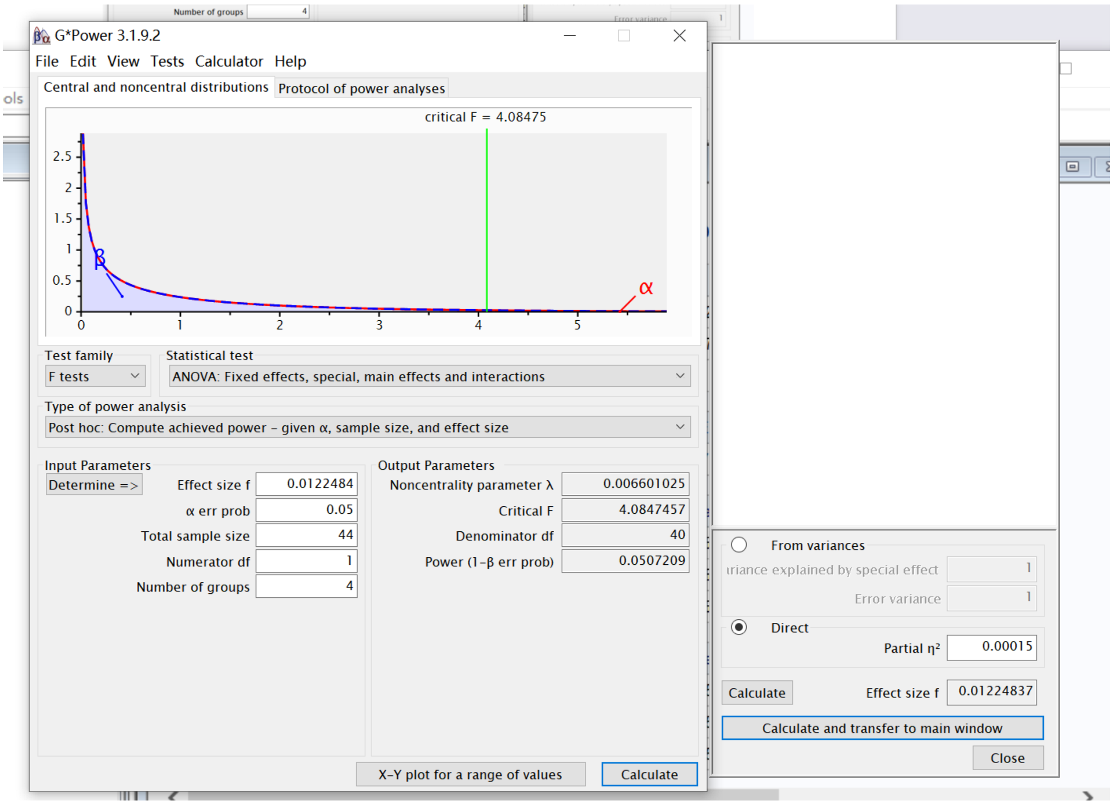
G*Power 3.1.9.2
File Edit View Tests Calculator Help
Central and noncentral distributions
Protocol of power analyses
critical F = 4.08475
Test family
F tests
Statistical test
ANOVA: Fixed effects, special, main effects and interactions
Type of power analysis
Post hoc: Compute achieved power – given α, sample size, and effect size
Input Parameters
Determine =>
Effect size f 0.0122484
α err prob 0.05
Total sample size 44
Numerator df 1
Number of groups 4
Output Parameters
Noncentrality parameter λ 0.006601025
Critical F 4.0847457
Denominator df 40
Power (1−β err prob) 0.0507209
X-Y plot for a range of values
Calculate
From variances
Error variance 1
Direct
Partial η² 0.00015
Calculate
Effect size f 0.01224837
Calculate and transfer to main window
Close

N

A factor

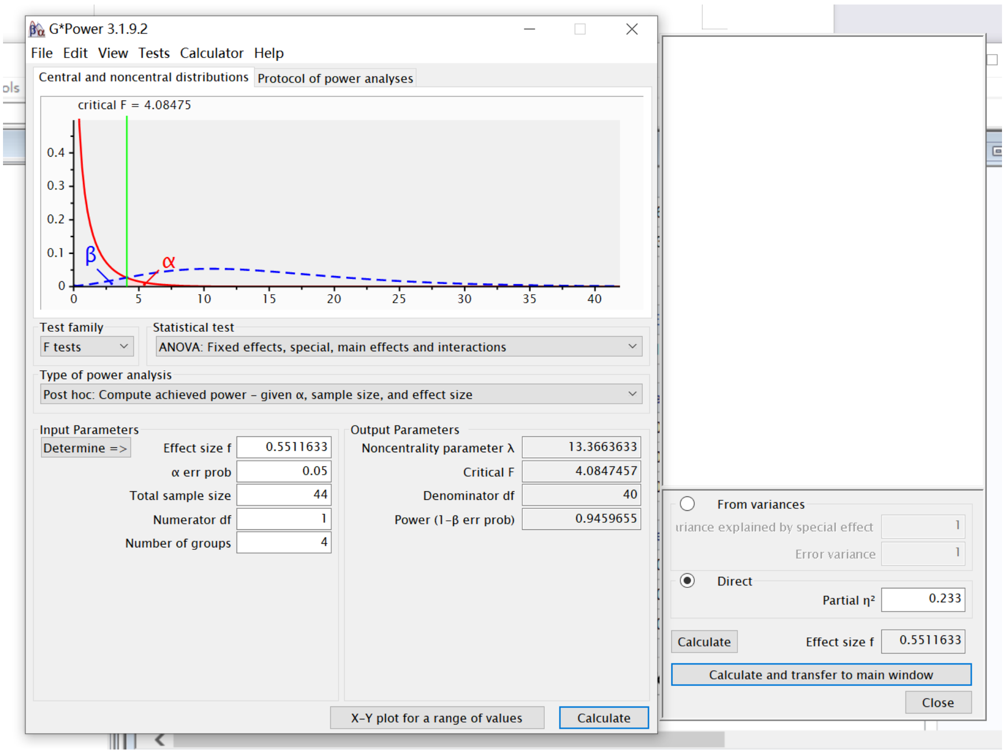
G*Power 3.1.9.2
File Edit View Tests Calculator Help
Central and noncentral distributions
Protocol of power analyses
critical F = 4.08475
Test family
F tests
Statistical test
ANOVA: Fixed effects, special, main effects and interactions
Type of power analysis
Post hoc: Compute achieved power – given α, sample size, and effect size
Input Parameters
Determine =>
Effect size f 0.5511633
α err prob 0.05
Total sample size 44
Numerator df 1
Number of groups 4
Output Parameters
Noncentrality parameter λ 13.3663633
Critical F 4.0847457
Denominator df 40
Power (1−β err prob) 0.9459655
X-Y plot for a range of values
Calculate
From variances
Error variance 1
Direct
Partial η² 0.233
Calculate
Effect size f 0.5511633
Calculate and transfer to main window
Close

B factor

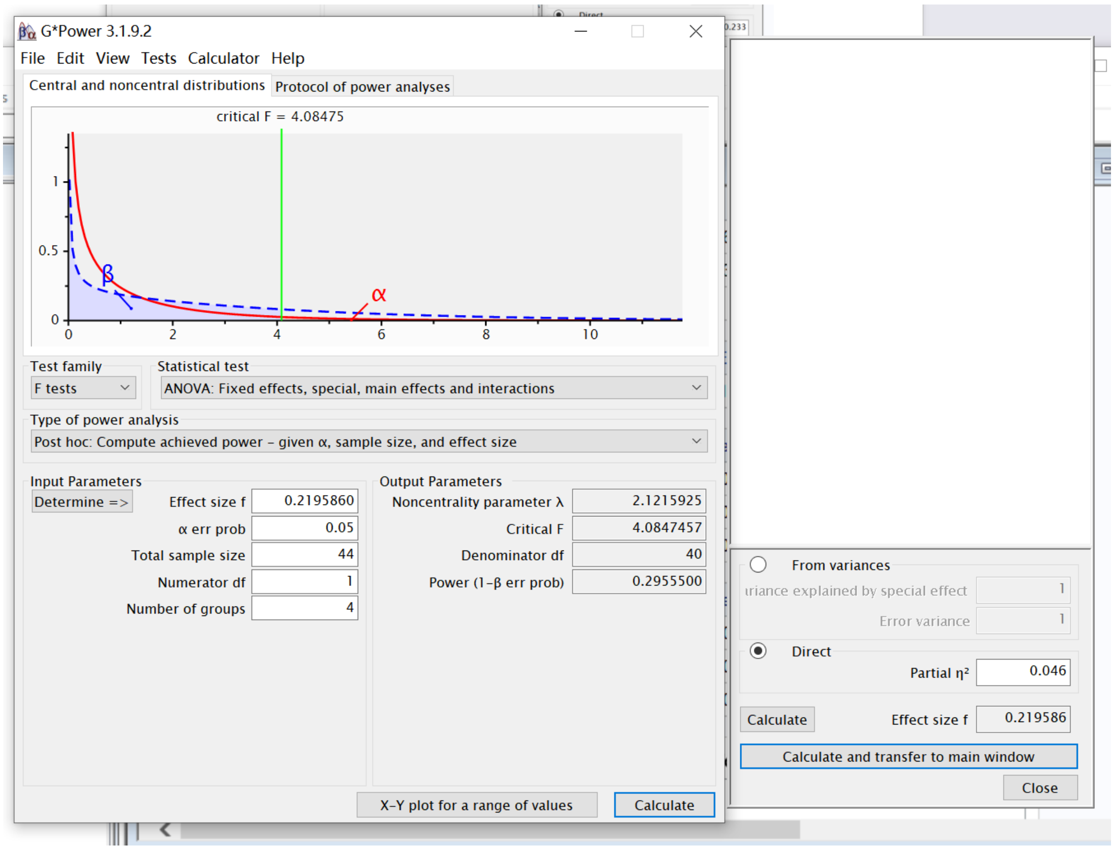
G*Power 3.1.9.2
File Edit View Tests Calculator Help
Central and noncentral distributions
Protocol of power analyses
critical F = 4.08475
Test family
F tests
Statistical test
ANOVA: Fixed effects, special, main effects and interactions
Type of power analysis
Post hoc: Compute achieved power – given α, sample size, and effect size
Input Parameters
Determine =>
Effect size f 0.2195860
α err prob 0.05
Total sample size 44
Numerator df 1
Number of groups 4
Output Parameters
Noncentrality parameter λ 2.1215925
Critical F 4.0847457
Denominator df 40
Power (1-β err prob) 0.2955500
X-Y plot for a range of values
Calculate
From variances
Error variance 1
Direct
Partial η² 0.046
Calculate
Effect size f 0.219586
Calculate and transfer to main window
Close

A ×B

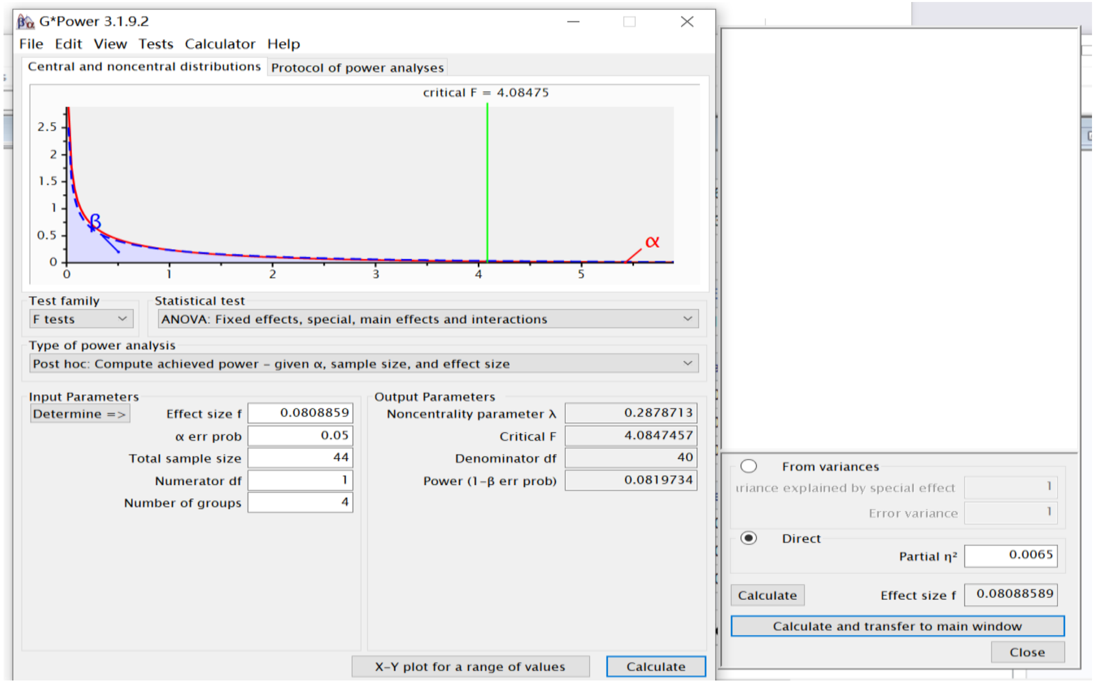
G*Power 3.1.9.2
File Edit View Tests Calculator Help
Central and noncentral distributions
Protocol of power analyses
critical F = 4.08475
Test family
F tests
Statistical test
ANOVA: Fixed effects, special, main effects and interactions
Type of power analysis
Post hoc: Compute achieved power – given α, sample size, and effect size
Input Parameters
Determine =>
Effect size f 0.0808859
α err prob 0.05
Total sample size 44
Numerator df 1
Number of groups 4
Output Parameters
Noncentrality parameter λ 0.2878713
Critical F 4.0847457
Denominator df 40
Power (1-β err prob) 0.0819734
X-Y plot for a range of values
Calculate
From variances
Error variance 1
Direct
Partial η² 0.0065
Calculate
Effect size f 0.08088589
Calculate and transfer to main window
Close

E

A factor

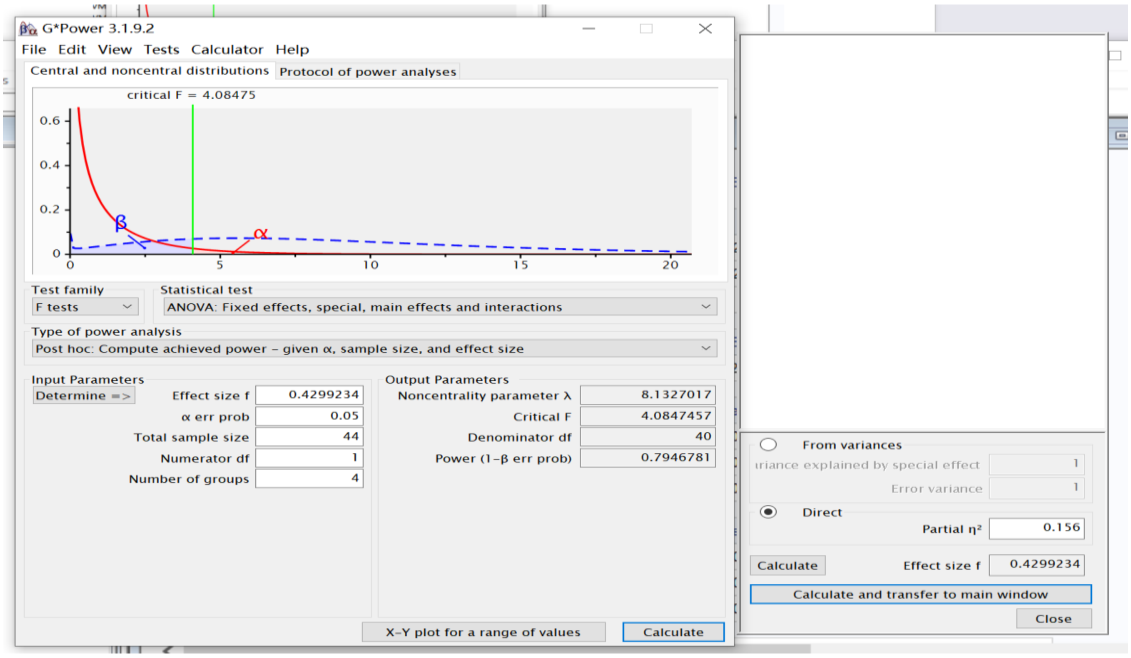

B factor

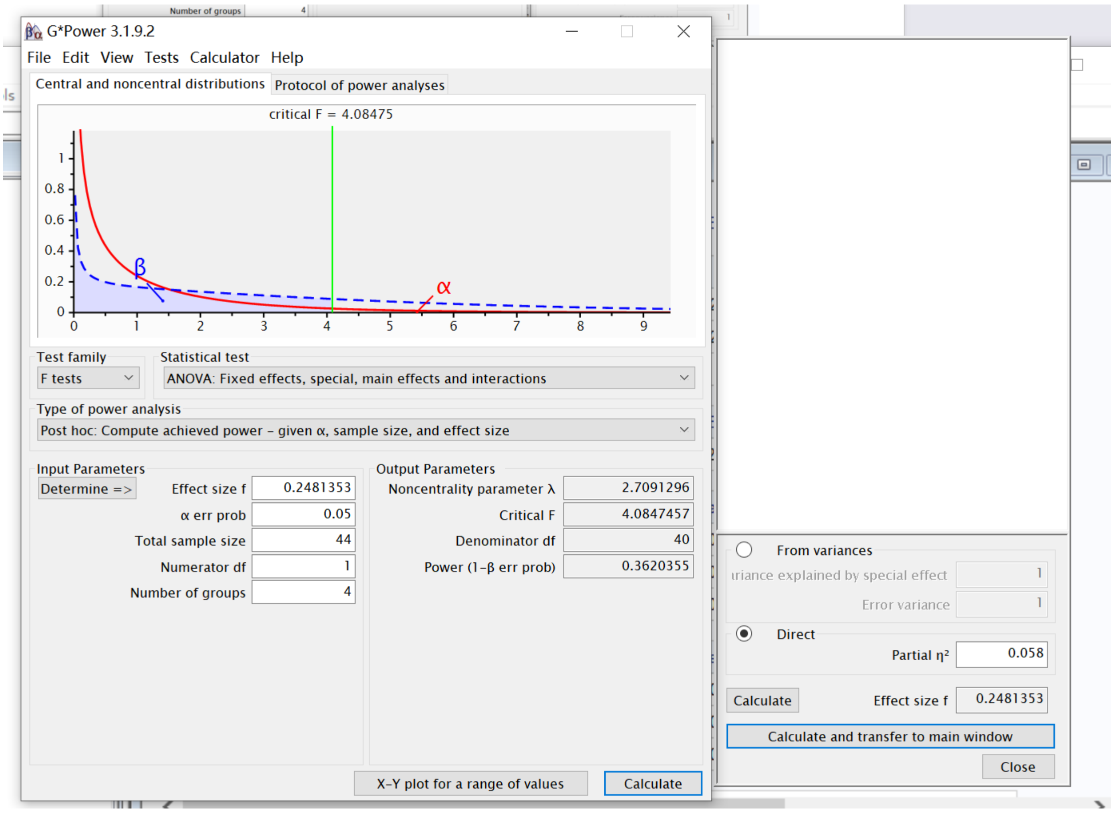

A ×B

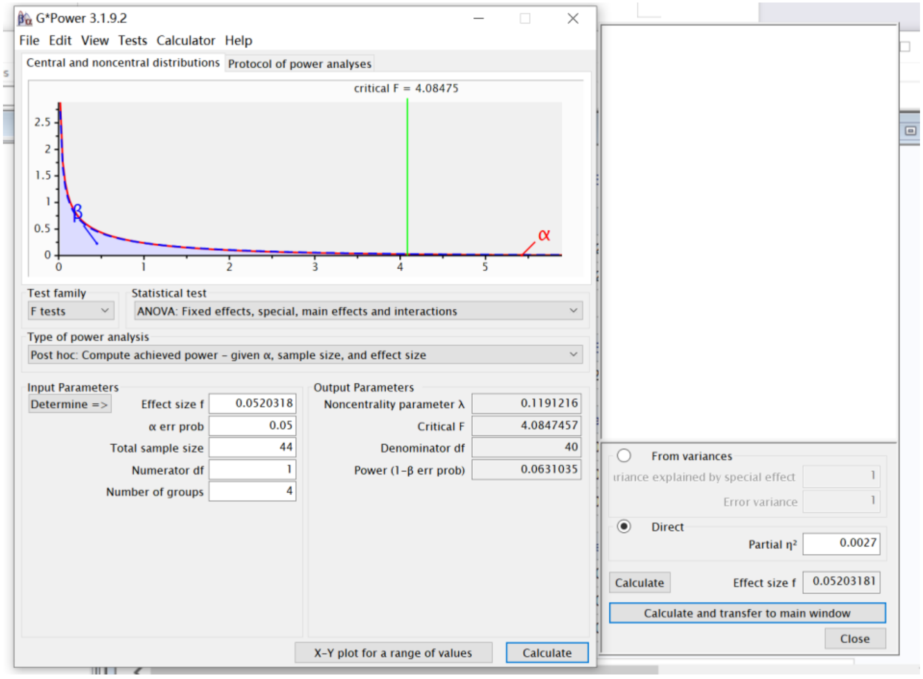
G*Power 3.1.9.2
File Edit View Tests Calculator Help
Central and noncentral distributions
Protocol of power analyses
critical F = 4.08475
Test family
F tests
Statistical test
ANOVA: Fixed effects, special, main effects and interactions
Type of power analysis
Post hoc: Compute achieved power – given α, sample size, and effect size
Input Parameters
Determine =>
Effect size f 0.0520318
α err prob 0.05
Total sample size 44
Numerator df 1
Number of groups 4
Output Parameters
Noncentrality parameter λ 0.1191216
Critical F 4.0847457
Denominator df 40
Power (1-β err prob) 0.0631035
X-Y plot for a range of values
Calculate
From variances
Error variance 1
Direct
Partial η² 0.0027
Calculate
Effect size f 0.05203181
Calculate and transfer to main window
Close

Exp. 2

A factor: EP corn

Factor B: PKM Diet

BW, WEEK 5

A factor

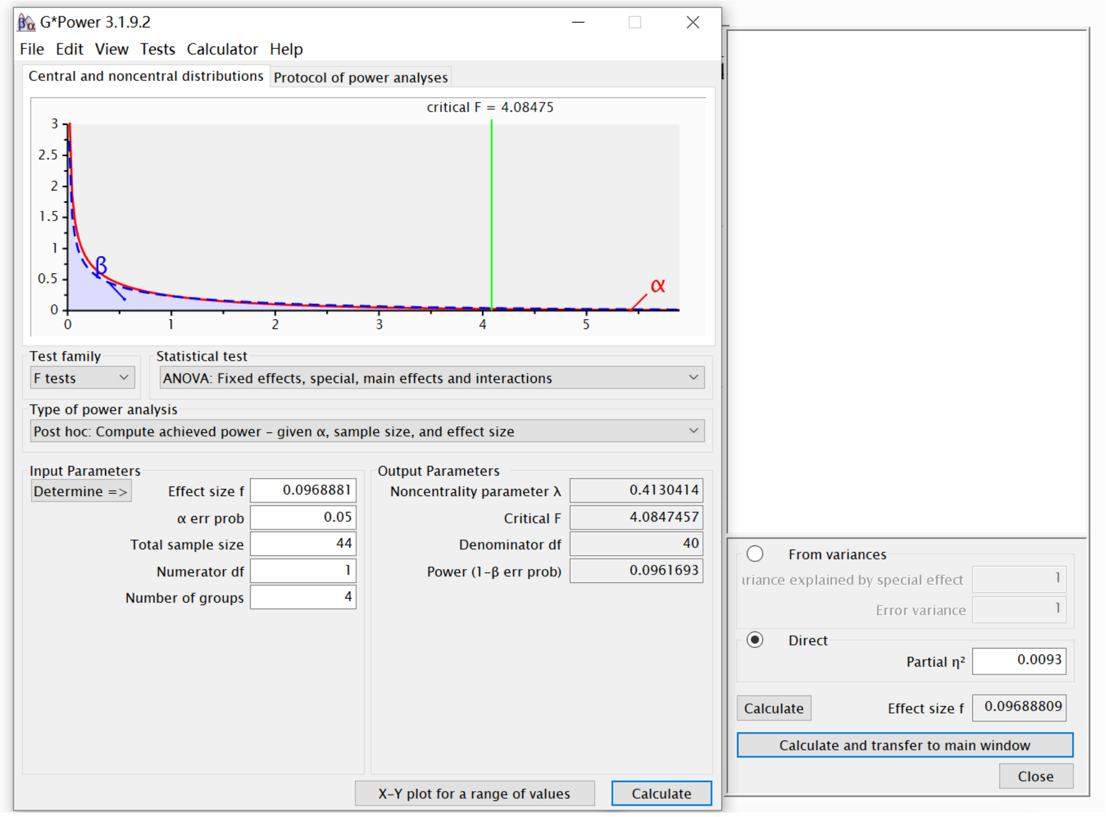

B factor

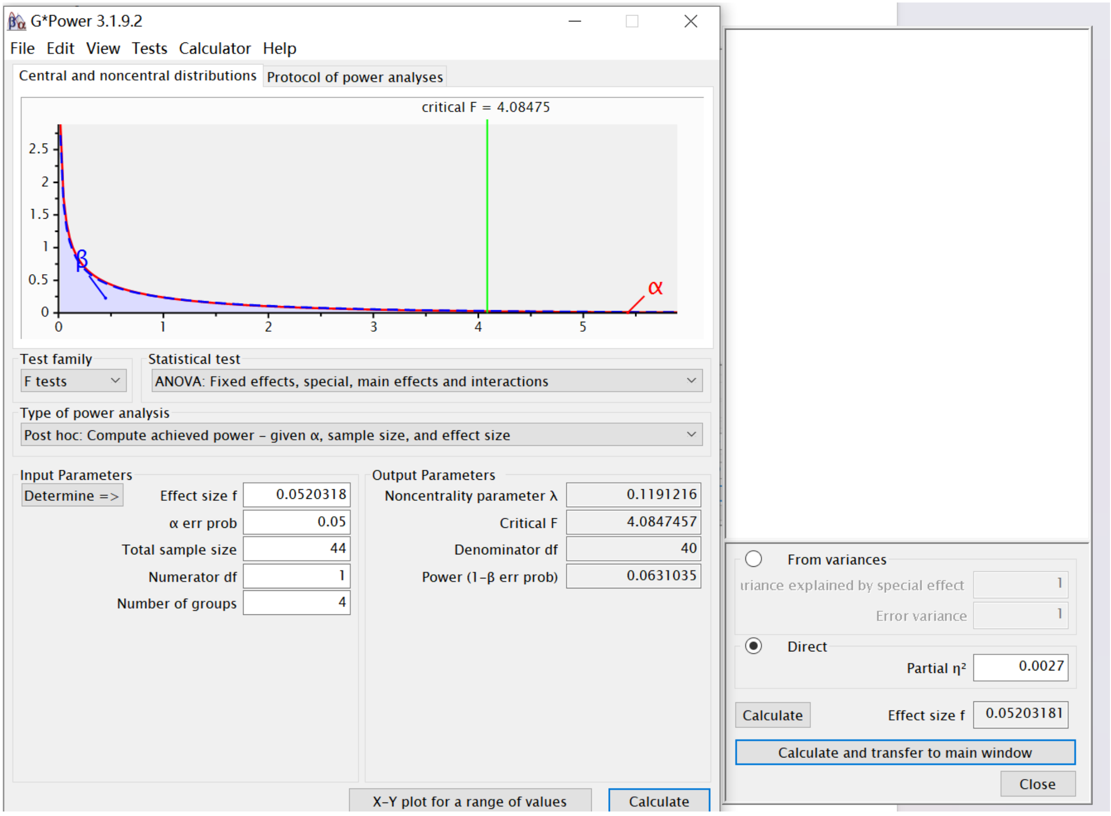
G*Power 3.1.9.2
File Edit View Tests Calculator Help
Central and noncentral distributions
Protocol of power analyses
critical F = 4.08475
Test family
F tests
Statistical test
ANOVA: Fixed effects, special, main effects and interactions
Type of power analysis
Post hoc: Compute achieved power – given α, sample size, and effect size
Input Parameters
Determine =>
Effect size f 0.0520318
α err prob 0.05
Total sample size 44
Numerator df 1
Number of groups 4
Output Parameters
Noncentrality parameter λ 0.1191216
Critical F 4.0847457
Denominator df 40
Power (1–β err prob) 0.0631035
X–Y plot for a range of values
Calculate
From variances
Error variance 1
Direct
Partial η² 0.0027
Calculate
Effect size f 0.05203181
Calculate and transfer to main window
Close

A ×B

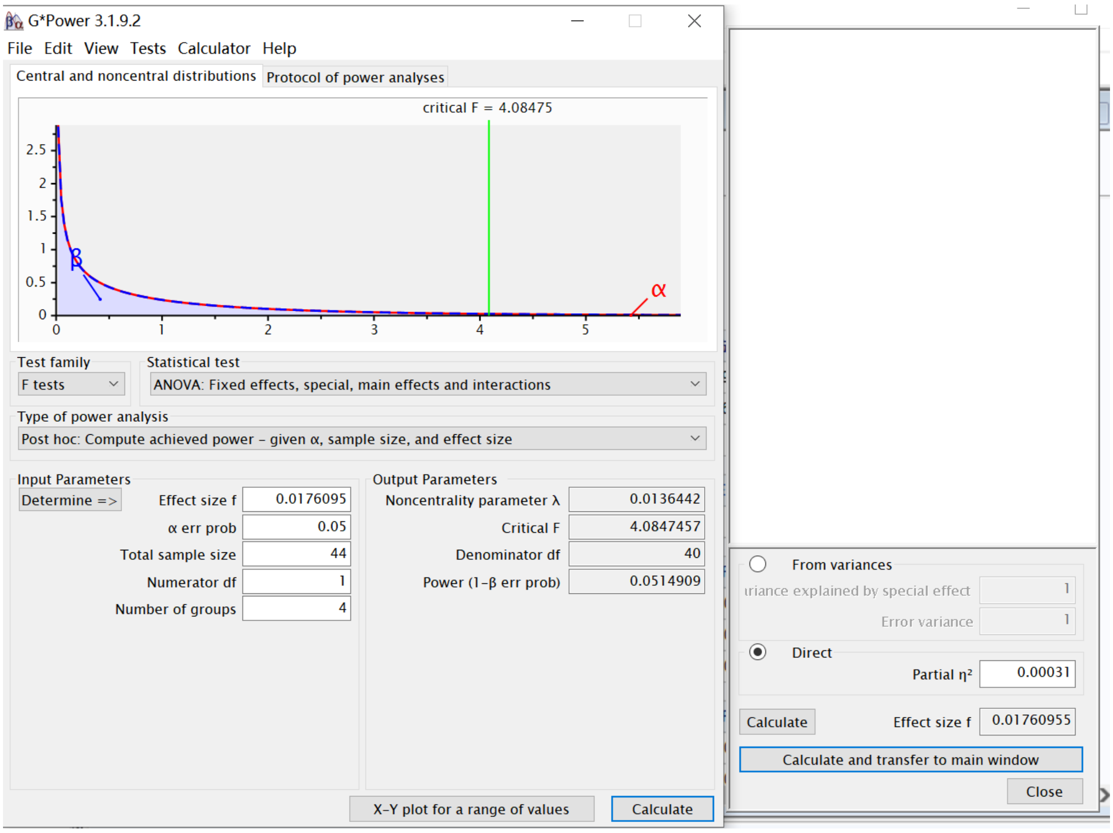
G*Power 3.1.9.2
File Edit View Tests Calculator Help
Central and noncentral distributions
Protocol of power analyses
critical F = 4.08475
Test family
F tests
Statistical test
ANOVA: Fixed effects, special, main effects and interactions
Type of power analysis
Post hoc: Compute achieved power – given α, sample size, and effect size
Input Parameters
Determine =>
Effect size f 0.0176095
α err prob 0.05
Total sample size 44
Numerator df 1
Number of groups 4
Output Parameters
Noncentrality parameter λ 0.0136442
Critical F 4.0847457
Denominator df 40
Power (1–β err prob) 0.0514909
X–Y plot for a range of values
Calculate
From variances
Error variance 1
Direct
Partial η² 0.00031
Calculate
Effect size f 0.01760955
Calculate and transfer to main window
Close

Week 10

BW

A factor

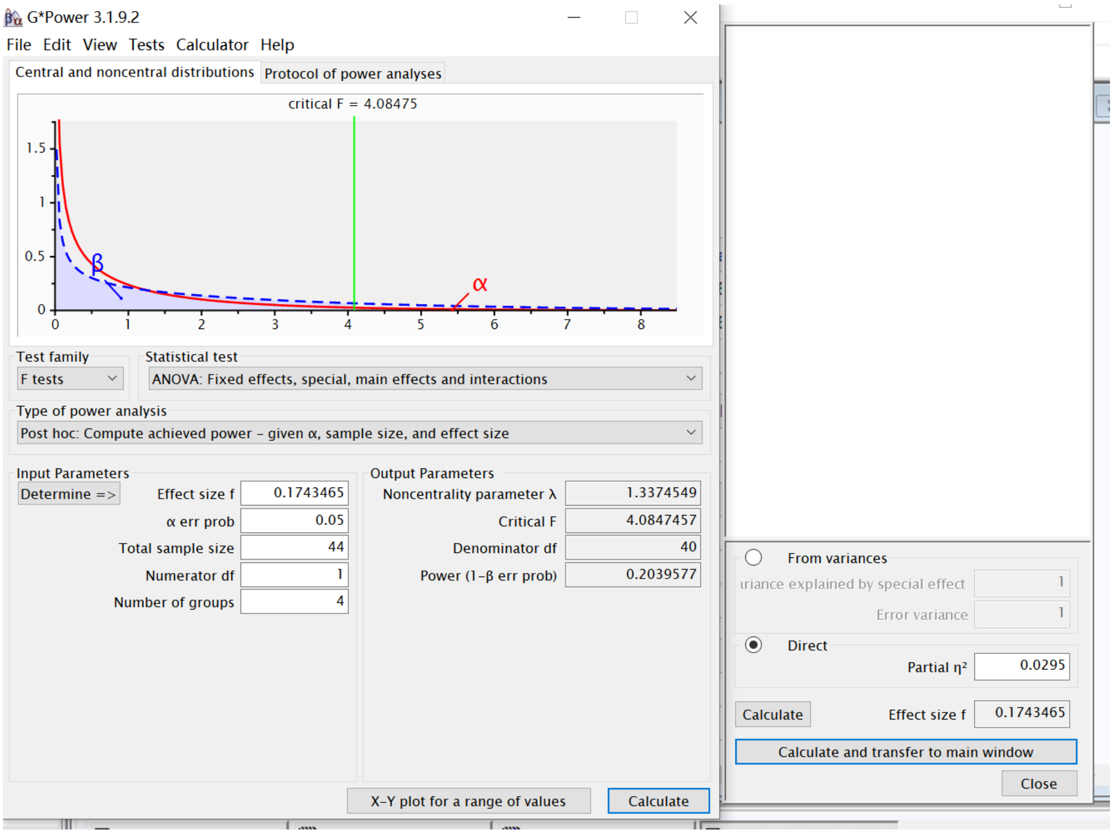

B factor

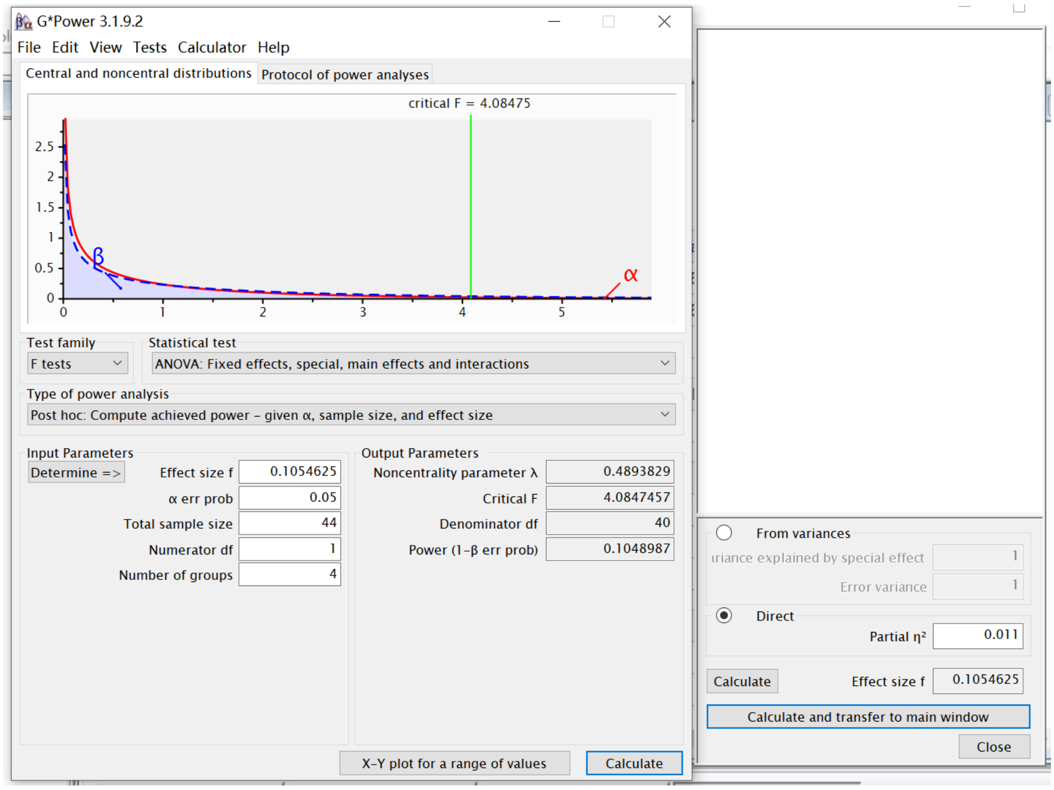

A ×B

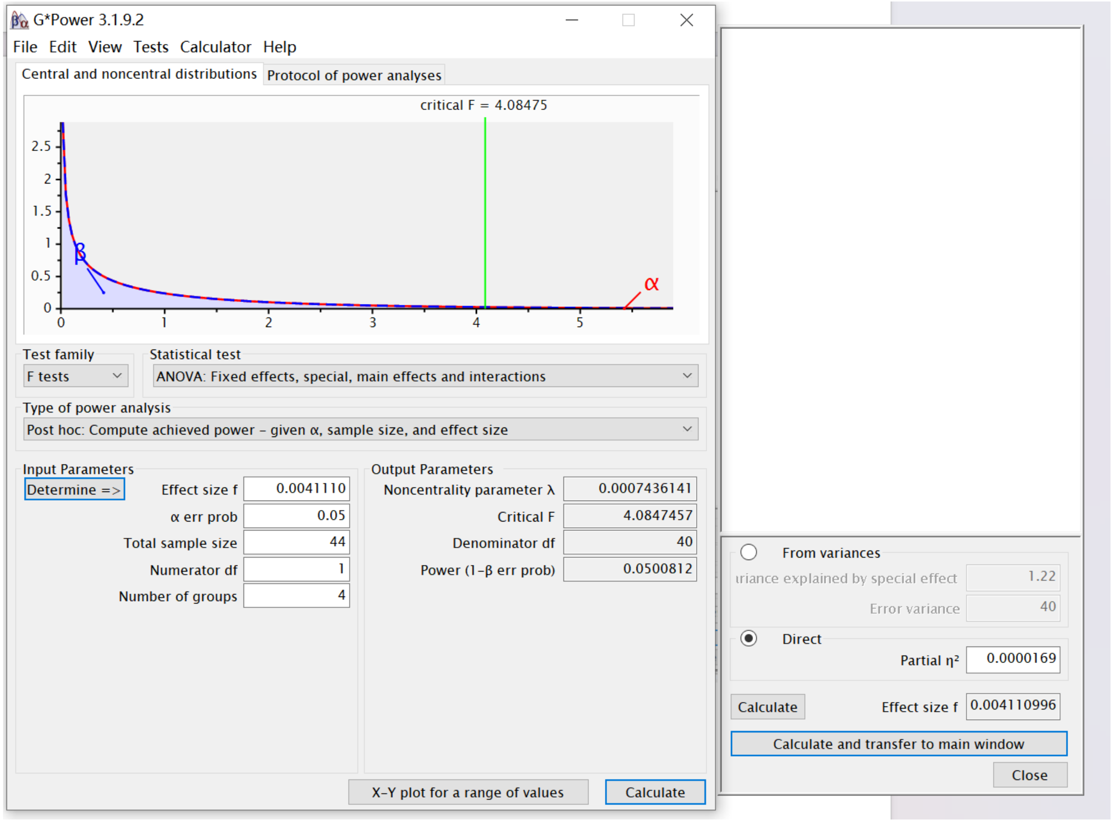

ADG week 5

A factor

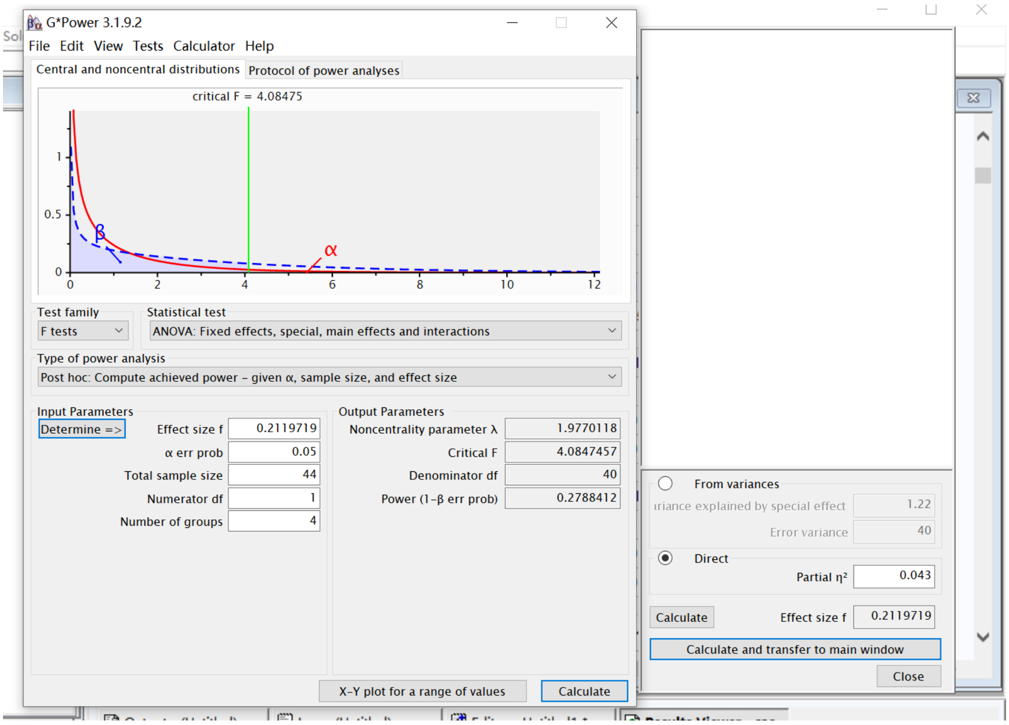

B factor

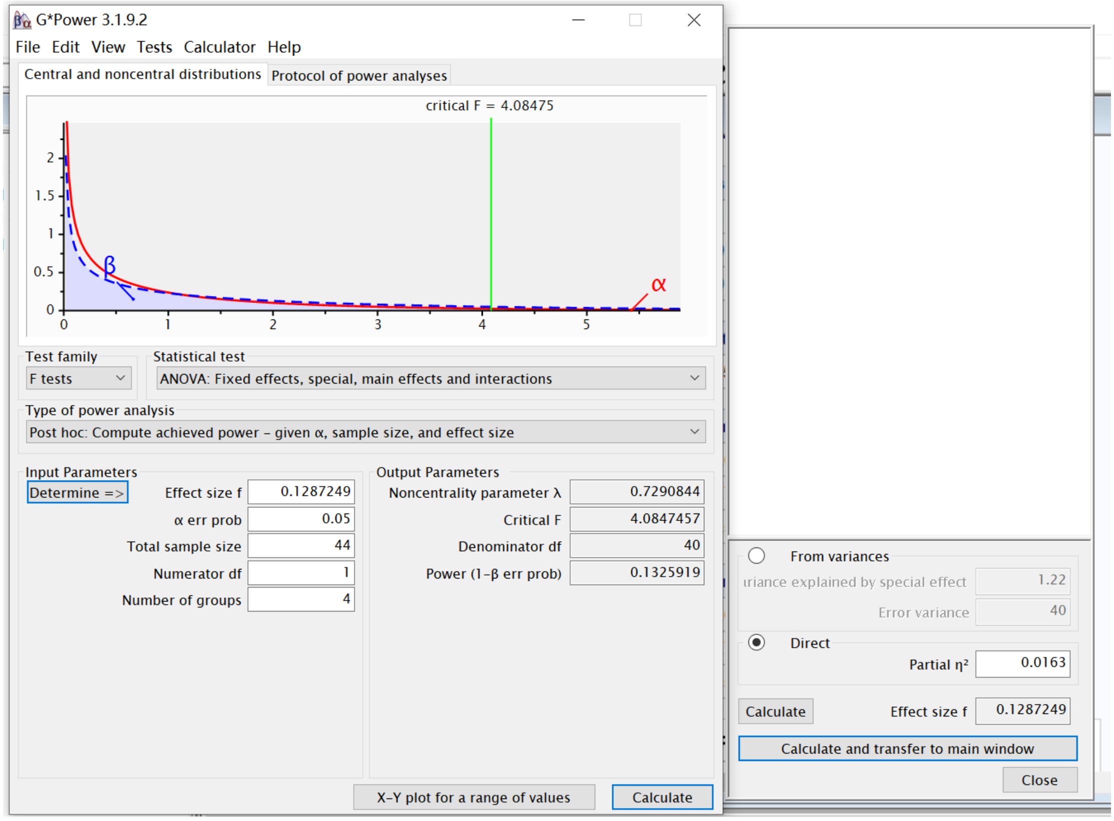
G*Power 3.1.9.2
File Edit View Tests Calculator Help
Central and noncentral distributions
Protocol of power analyses
critical F = 4.08475
Test family
F tests
Statistical test
ANOVA: Fixed effects, special, main effects and interactions
Type of power analysis
Post hoc: Compute achieved power – given α, sample size, and effect size
Input Parameters
Determine =>
Effect size f 0.1287249
α err prob 0.05
Total sample size 44
Numerator df 1
Number of groups 4
Output Parameters
Noncentrality parameter λ 0.7290844
Critical F 4.0847457
Denominator df 40
Power (1-β err prob) 0.1325919
X-Y plot for a range of values
Calculate
From variances
Error variance 40
Direct
Partial η² 0.0163
Calculate
Effect size f 0.1287249
Calculate and transfer to main window
Close

A×B

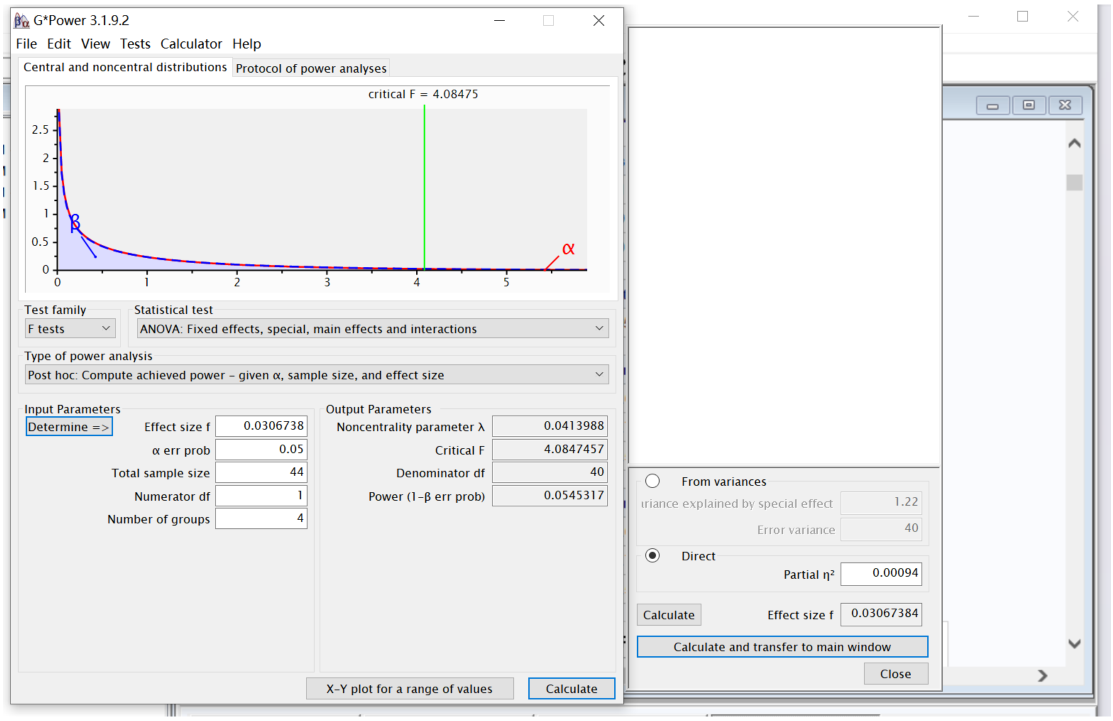
G*Power 3.1.9.2
File Edit View Tests Calculator Help
Central and noncentral distributions
Protocol of power analyses
critical F = 4.08475
Test family
F tests
Statistical test
ANOVA: Fixed effects, special, main effects and interactions
Type of power analysis
Post hoc: Compute achieved power – given α, sample size, and effect size
Input Parameters
Determine =>
Effect size f 0.0306738
α err prob 0.05
Total sample size 44
Numerator df 1
Number of groups 4
Output Parameters
Noncentrality parameter λ 0.0413988
Critical F 4.0847457
Denominator df 40
Power (1-β err prob) 0.0545317
X-Y plot for a range of values
Calculate
From variances
Error variance 40
Direct
Partial η² 0.00094
Calculate
Effect size f 0.03067384
Calculate and transfer to main window
Close

ADG week 10

A factor

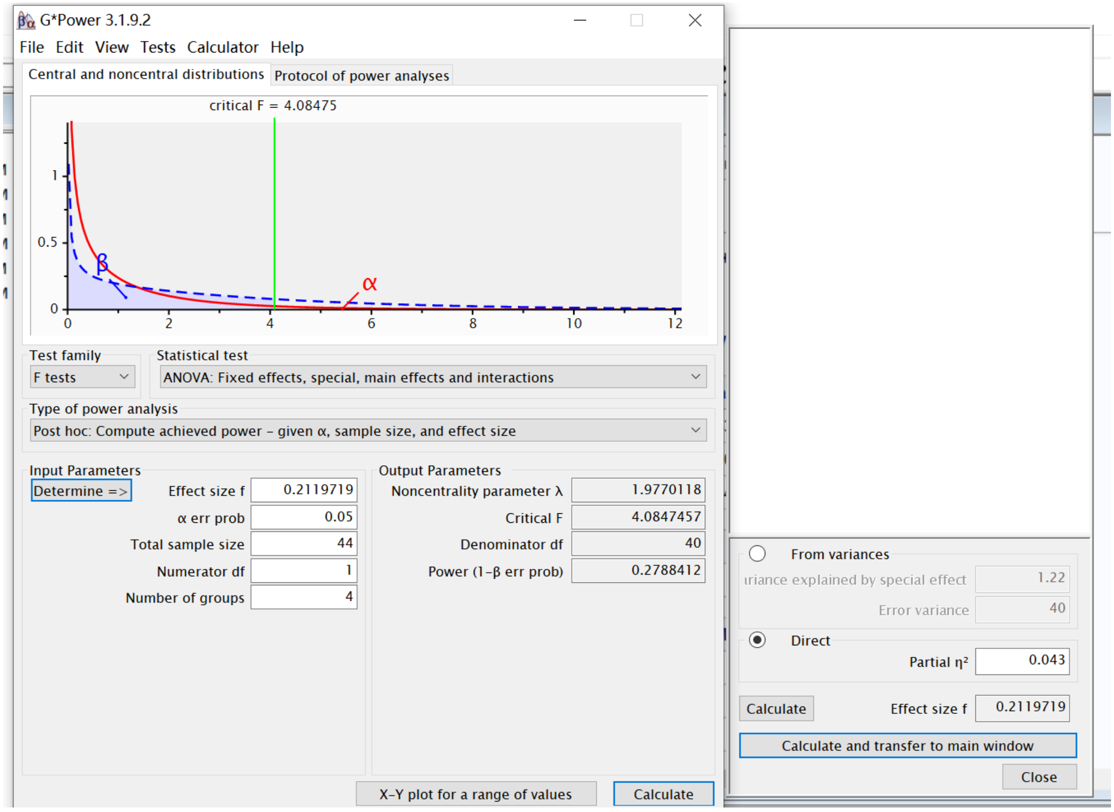

B factor

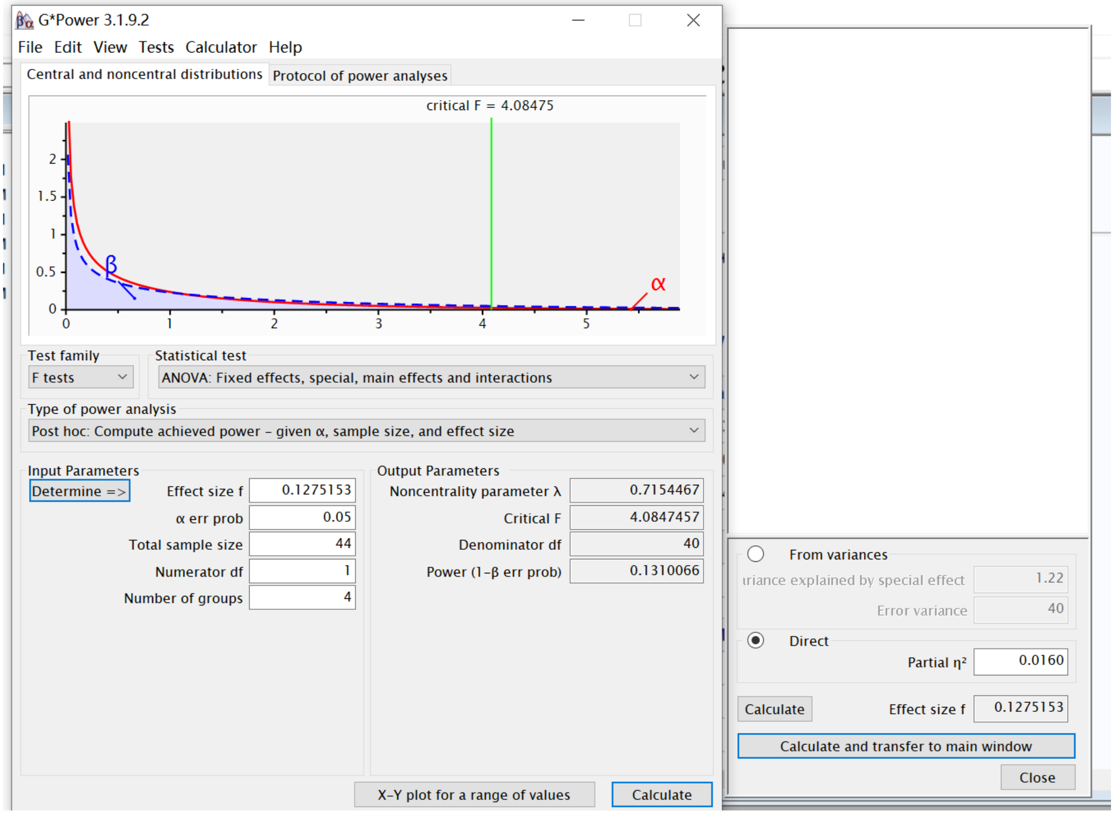

A ×B

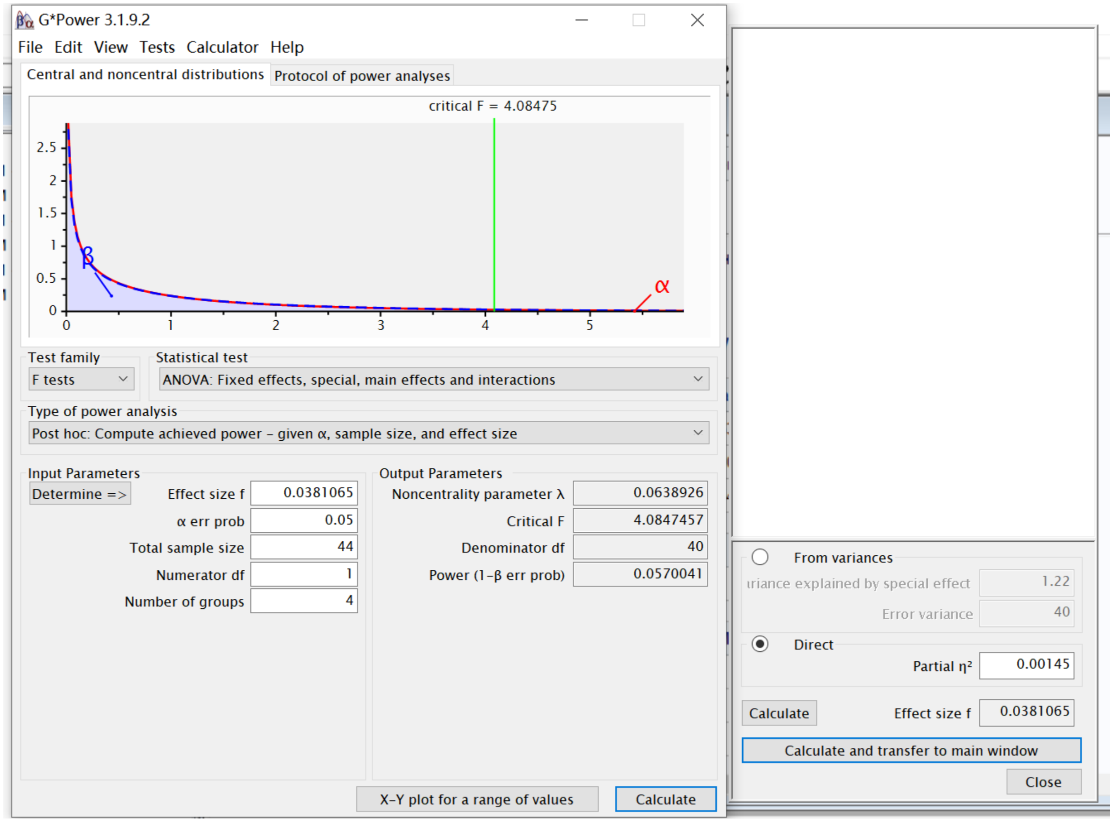

ADFI week 5

A factor

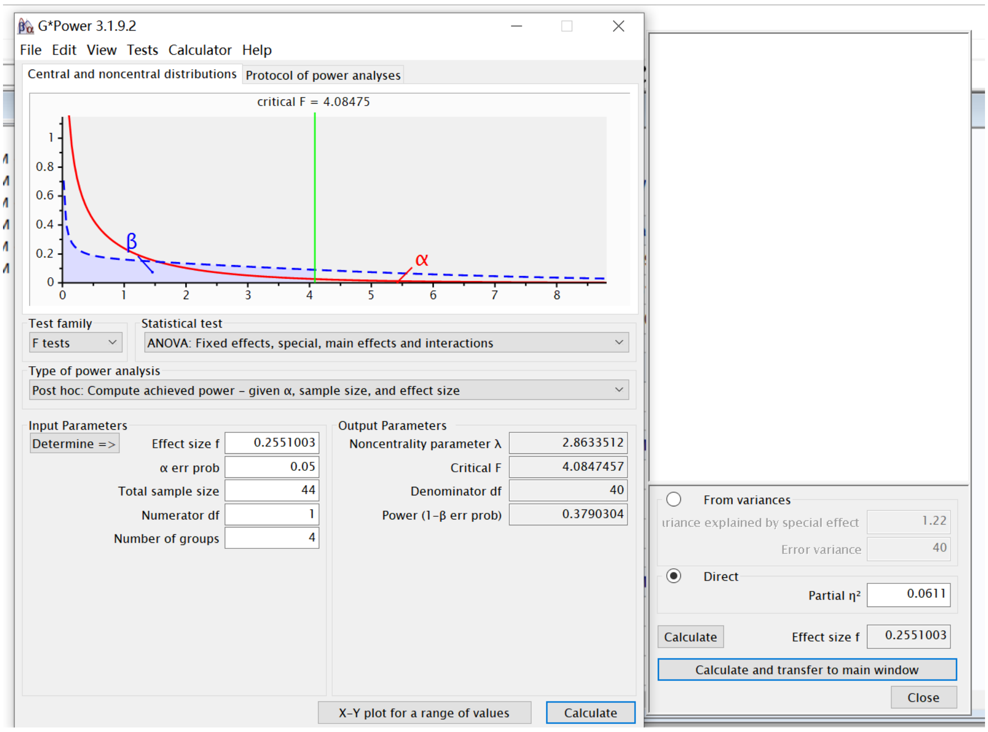

B factor

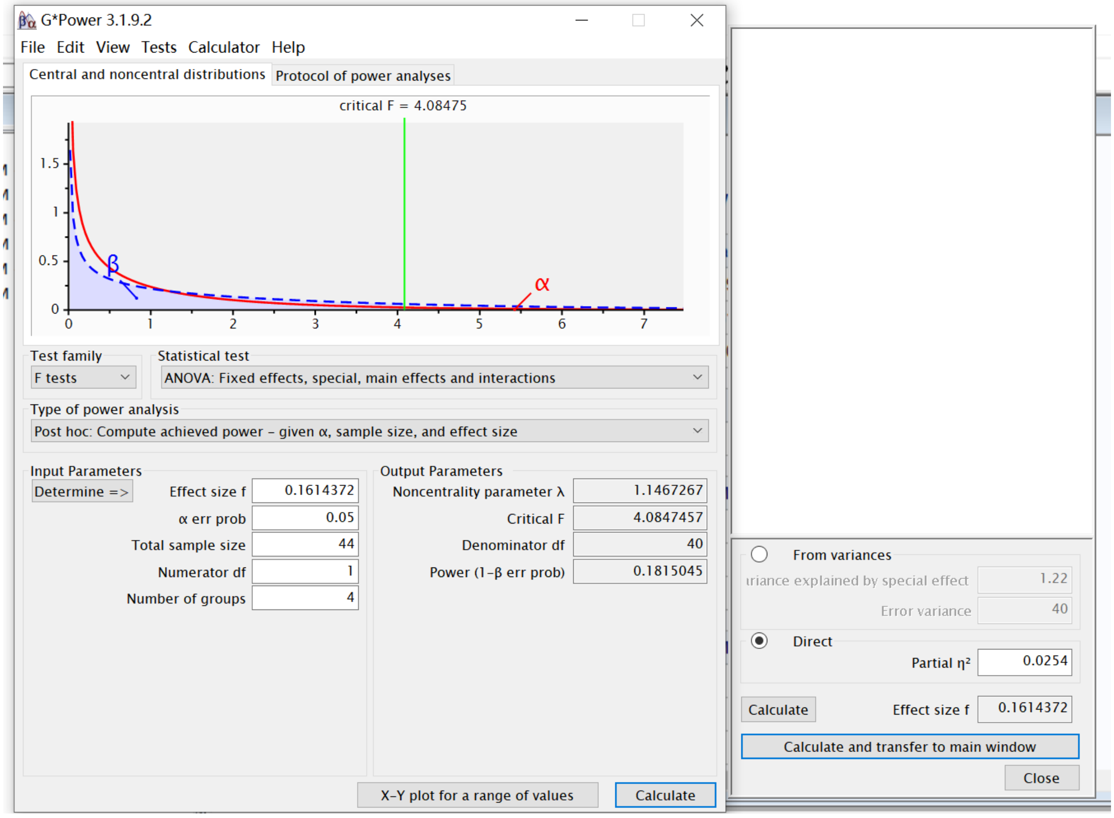

G*Power 3.1.9.2
File Edit View Tests Calculator Help
Central and noncentral distributions
Protocol of power analyses
critical F = 4.08475
Test family
F tests
Statistical test
ANOVA: Fixed effects, special, main effects and interactions
Type of power analysis
Post hoc: Compute achieved power – given α, sample size, and effect size
Input Parameters
Determine =>
Effect size f 0.1614372
α err prob 0.05
Total sample size 44
Numerator df 1
Number of groups 4
Output Parameters
Noncentrality parameter λ 1.1467267
Critical F 4.0847457
Denominator df 40
Power (1–β err prob) 0.1815045
X–Y plot for a range of values
Calculate
From variances
Variance explained by special effect 1.22
Error variance 40
Direct
Partial η² 0.0254
Calculate
Effect size f 0.1614372
Calculate and transfer to main window
Close

A ×B

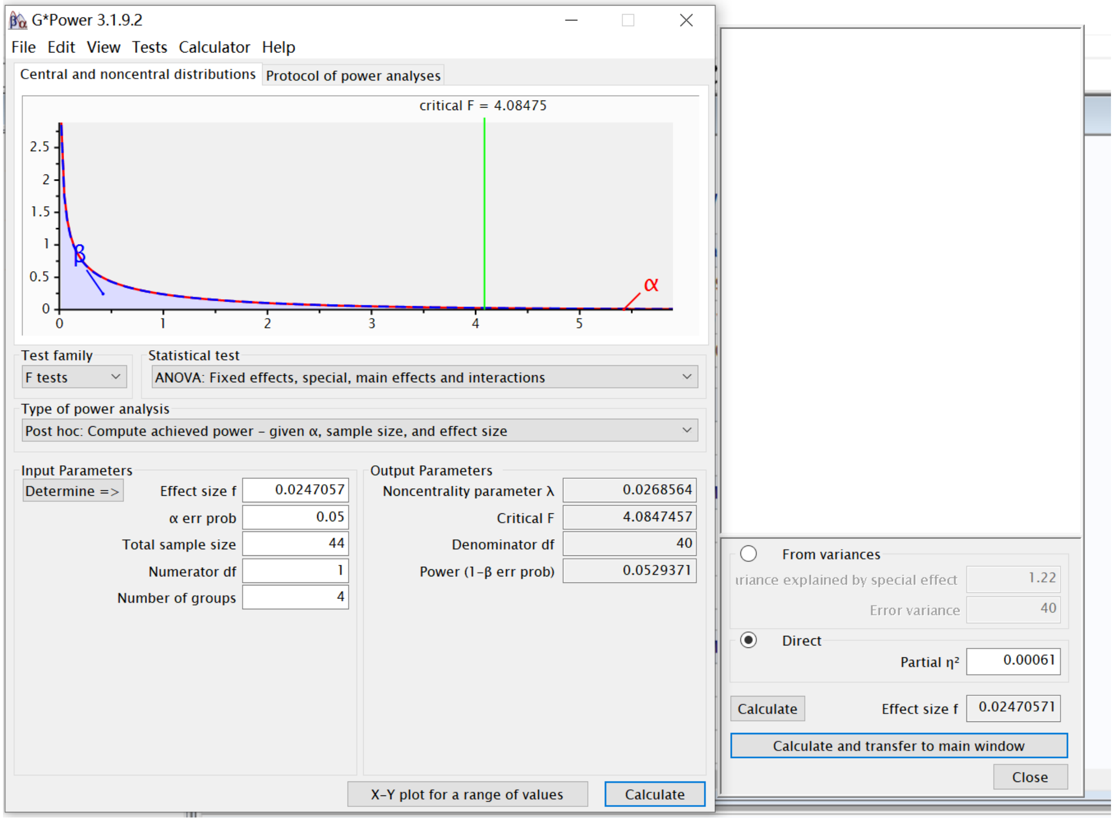

G*Power 3.1.9.2
File Edit View Tests Calculator Help
Central and noncentral distributions
Protocol of power analyses
critical F = 4.08475
Test family
F tests
Statistical test
ANOVA: Fixed effects, special, main effects and interactions
Type of power analysis
Post hoc: Compute achieved power – given α, sample size, and effect size
Input Parameters
Determine =>
Effect size f 0.0247057
α err prob 0.05
Total sample size 44
Numerator df 1
Number of groups 4
Output Parameters
Noncentrality parameter λ 0.0268564
Critical F 4.0847457
Denominator df 40
Power (1–β err prob) 0.0529371
X–Y plot for a range of values
Calculate
From variances
Variance explained by special effect 1.22
Error variance 40
Direct
Partial η² 0.00061
Calculate
Effect size f 0.02470571
Calculate and transfer to main window
Close

ADFI week 10

A factor

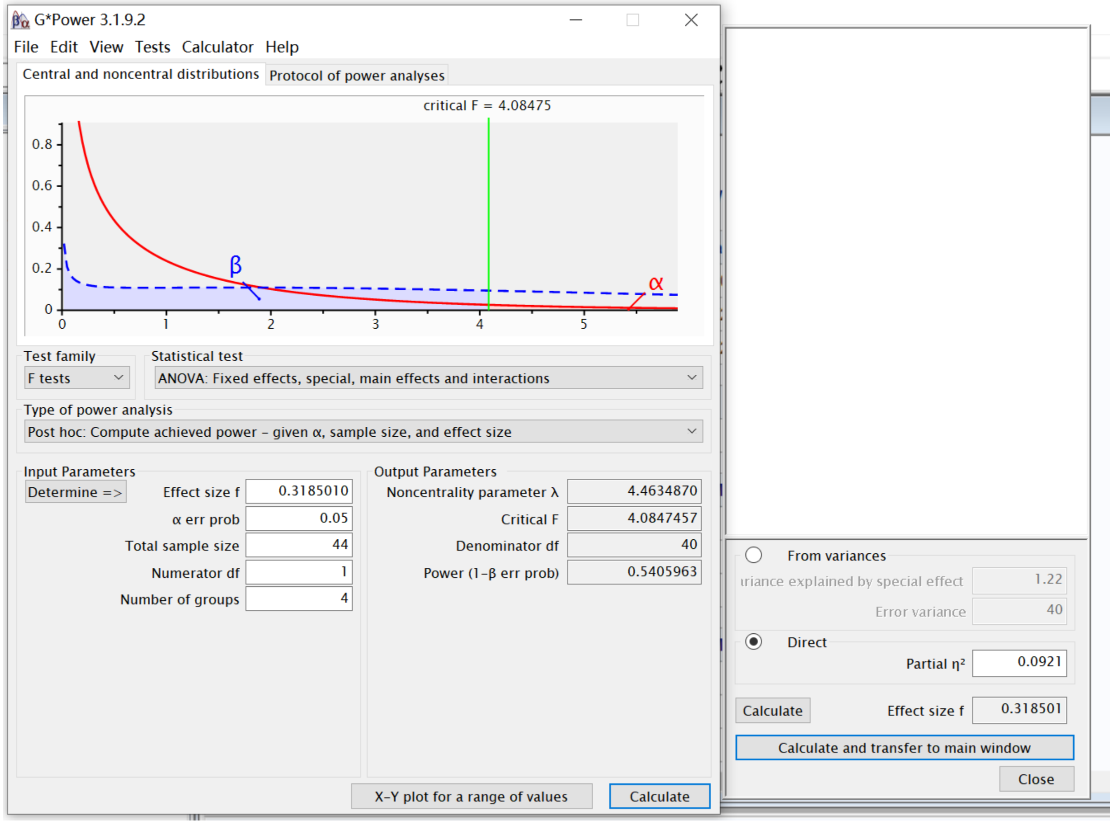

B factor

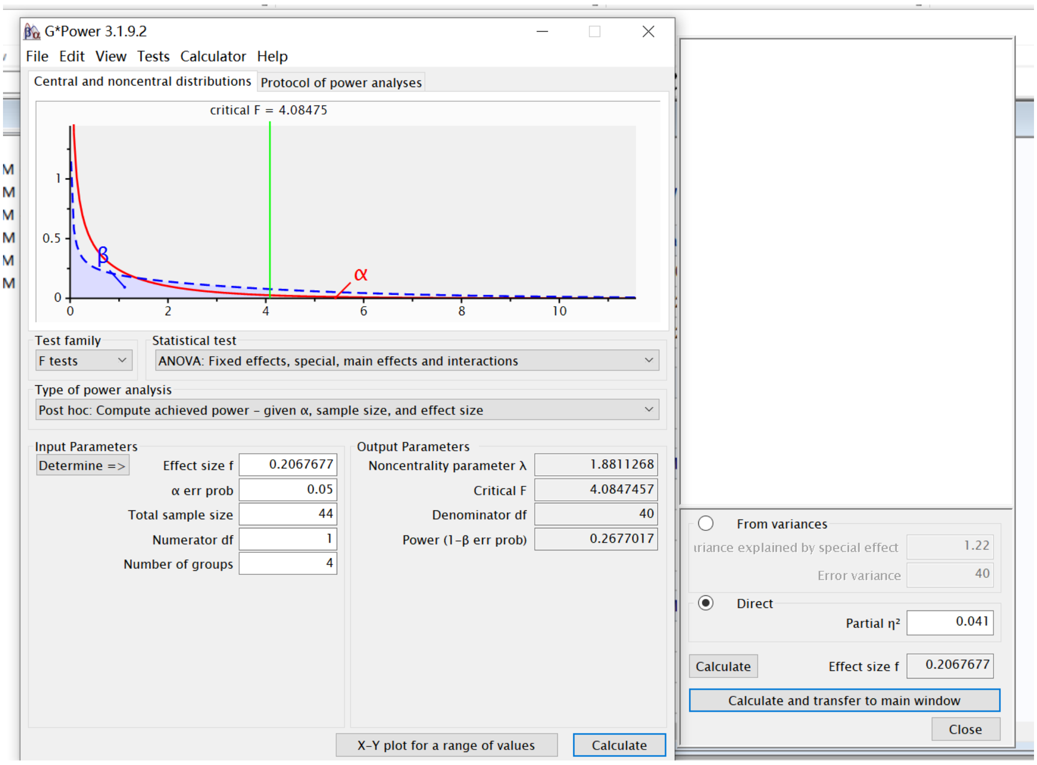

A ×B

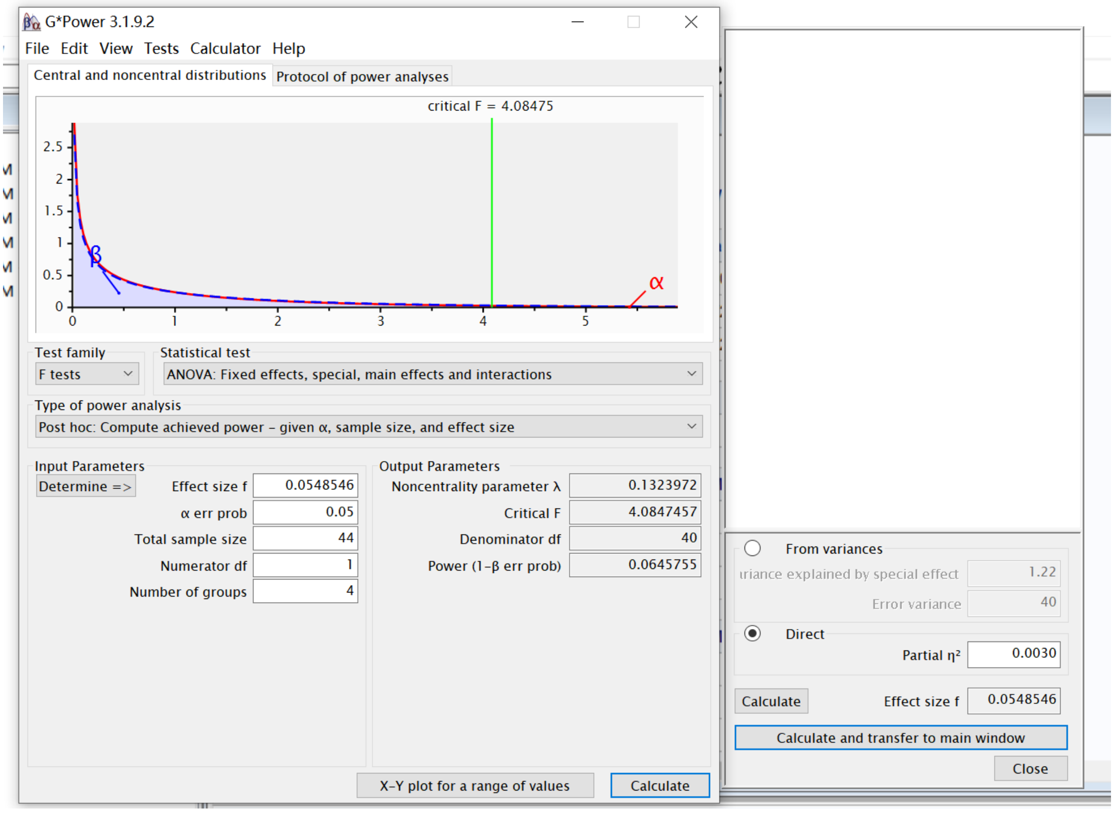

G:F week 5

A factor

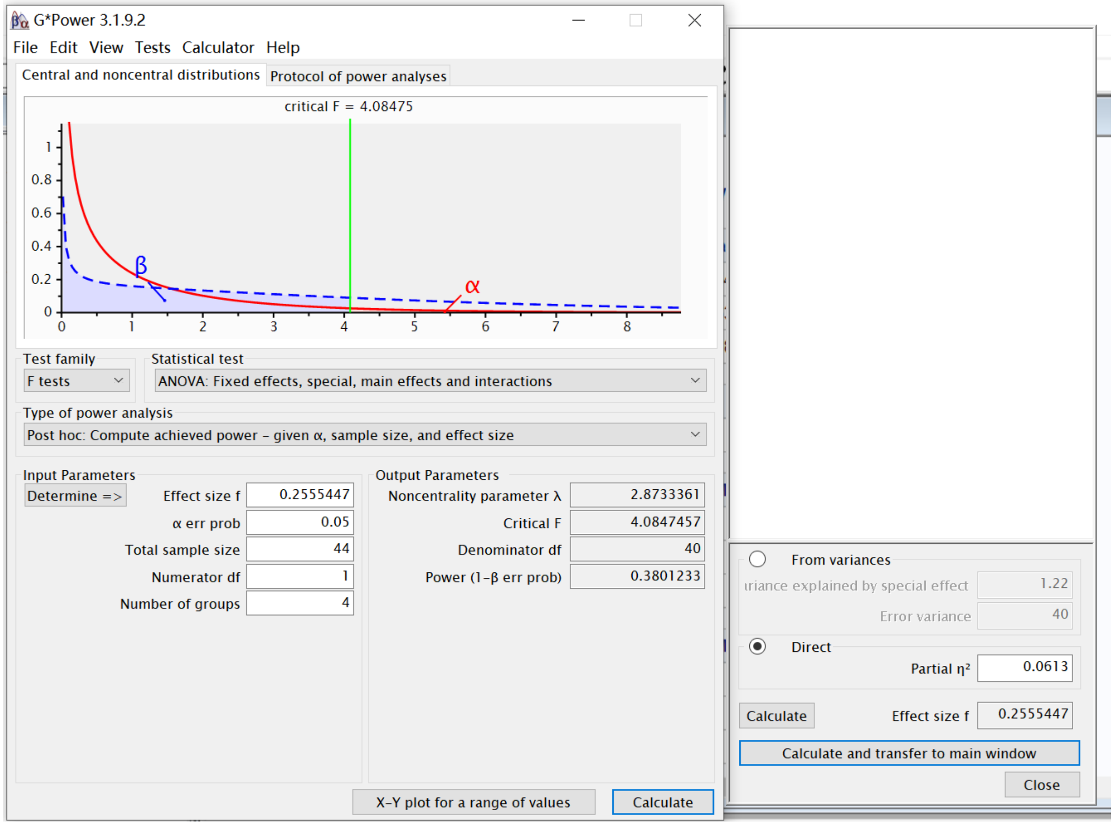

B factor

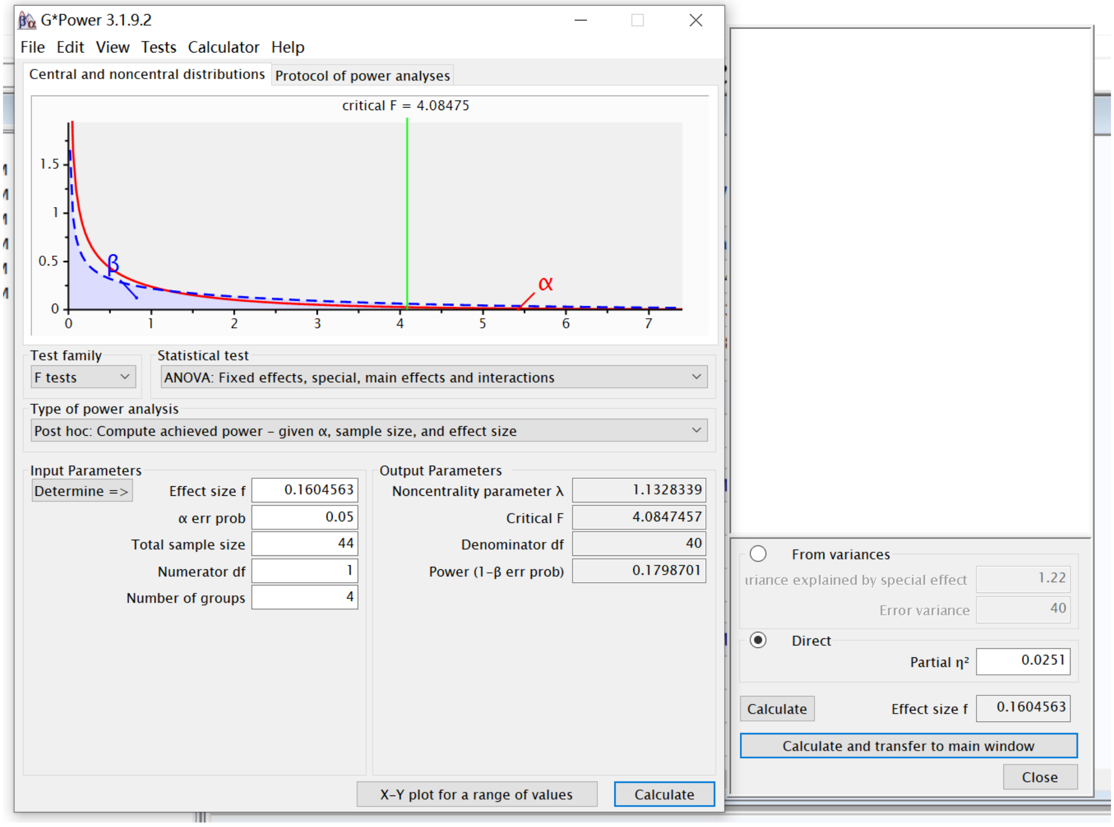

G*Power 3.1.9.2
File Edit View Tests Calculator Help
Central and noncentral distributions
Protocol of power analyses
critical F = 4.08475
Test family
F tests
Statistical test
ANOVA: Fixed effects, special, main effects and interactions
Type of power analysis
Post hoc: Compute achieved power – given α, sample size, and effect size
Input Parameters
Determine =>
Effect size f 0.1604563
α err prob 0.05
Total sample size 44
Numerator df 1
Number of groups 4
Output Parameters
Noncentrality parameter λ 1.1328339
Critical F 4.0847457
Denominator df 40
Power (1–β err prob) 0.1798701
X-Y plot for a range of values
Calculate
From variances
ıriance explained by special effect 1.22
Error variance 40
Direct
Partial η² 0.0251
Calculate
Effect size f 0.1604563
Calculate and transfer to main window
Close

A ×B

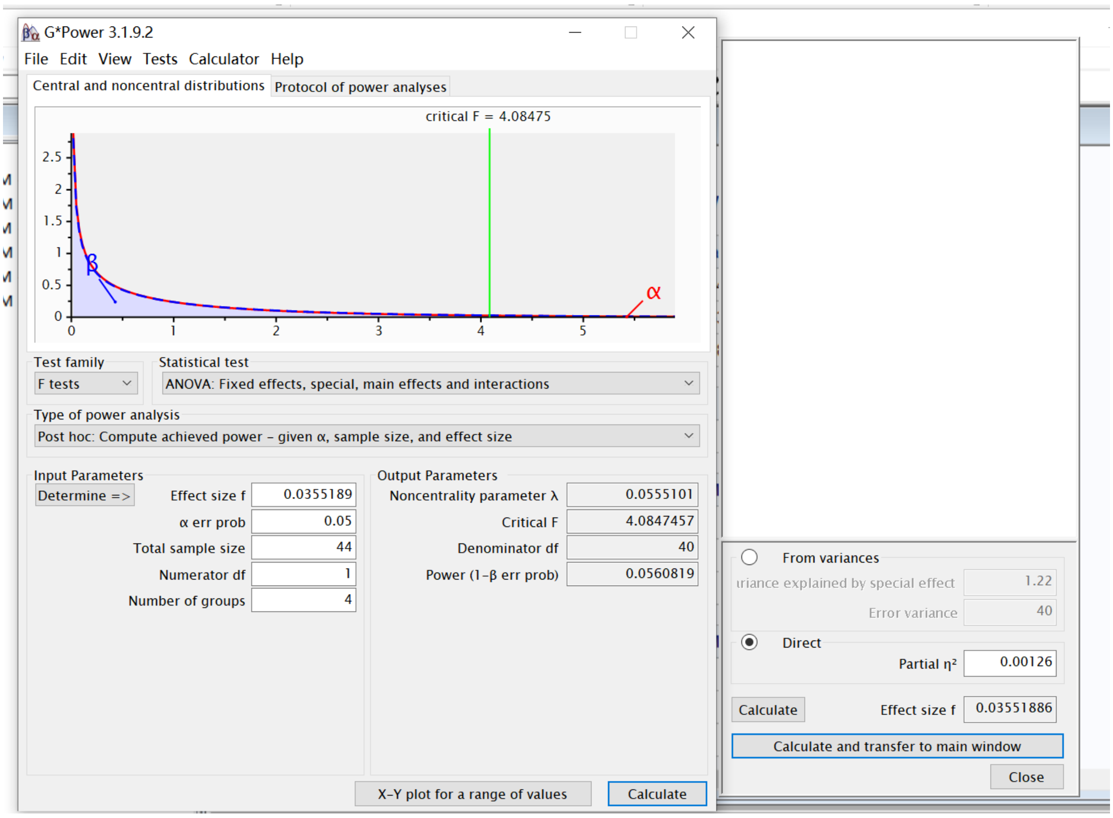

G*Power 3.1.9.2
File Edit View Tests Calculator Help
Central and noncentral distributions
Protocol of power analyses
critical F = 4.08475
Test family
F tests
Statistical test
ANOVA: Fixed effects, special, main effects and interactions
Type of power analysis
Post hoc: Compute achieved power – given α, sample size, and effect size
Input Parameters
Determine =>
Effect size f 0.0355189
α err prob 0.05
Total sample size 44
Numerator df 1
Number of groups 4
Output Parameters
Noncentrality parameter λ 0.0555101
Critical F 4.0847457
Denominator df 40
Power (1–β err prob) 0.0560819
X-Y plot for a range of values
Calculate
From variances
ıriance explained by special effect 1.22
Error variance 40
Direct
Partial η² 0.00126
Calculate
Effect size f 0.03551886
Calculate and transfer to main window
Close

G:F week 10

A factor

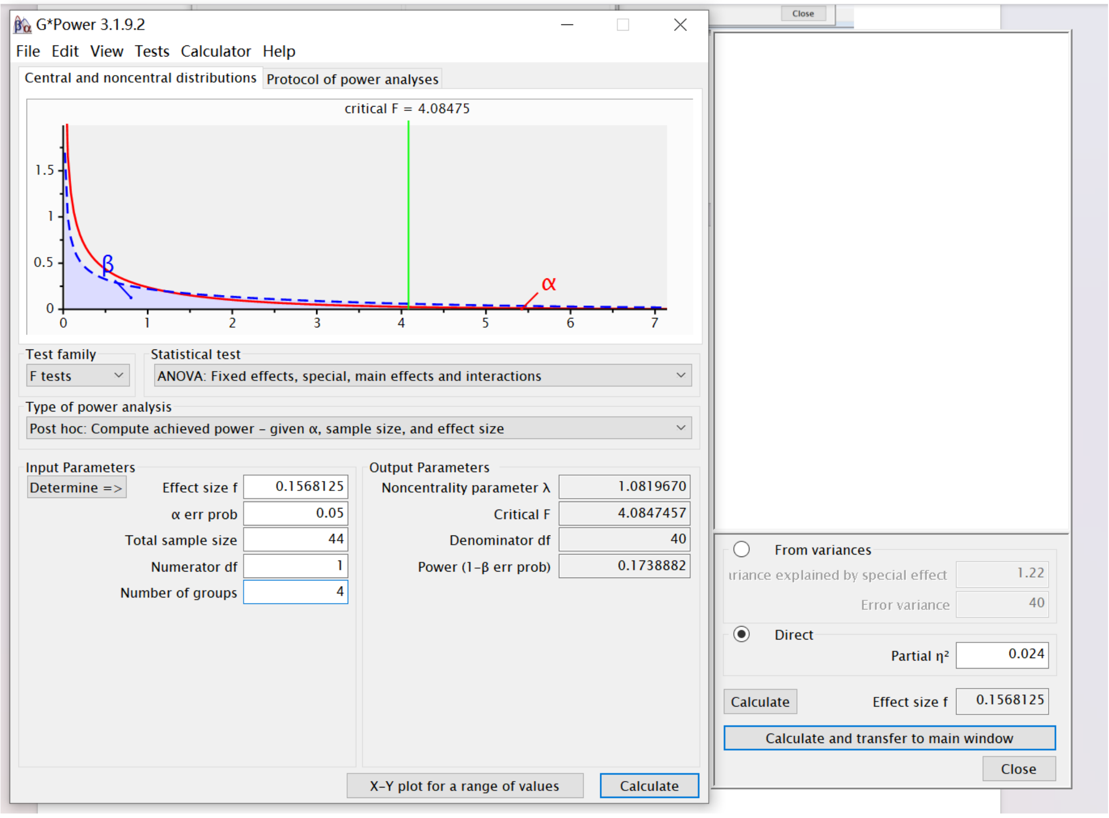

B factor

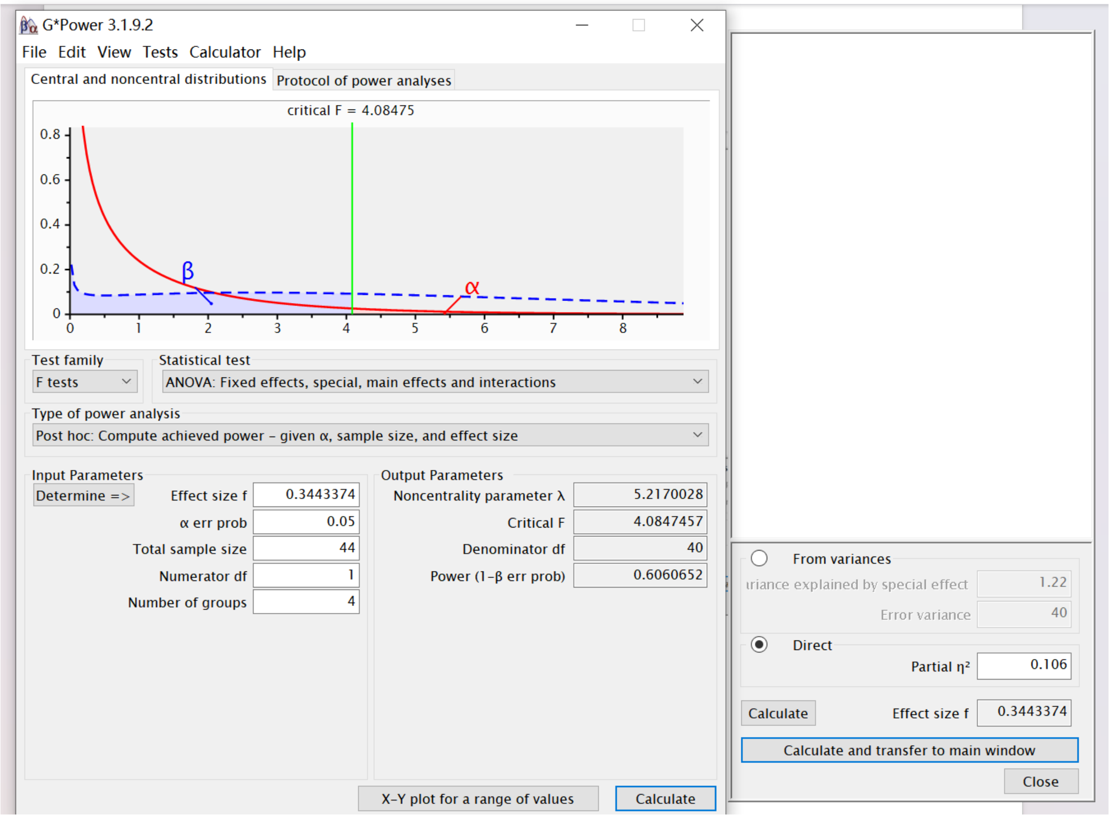

A ×B

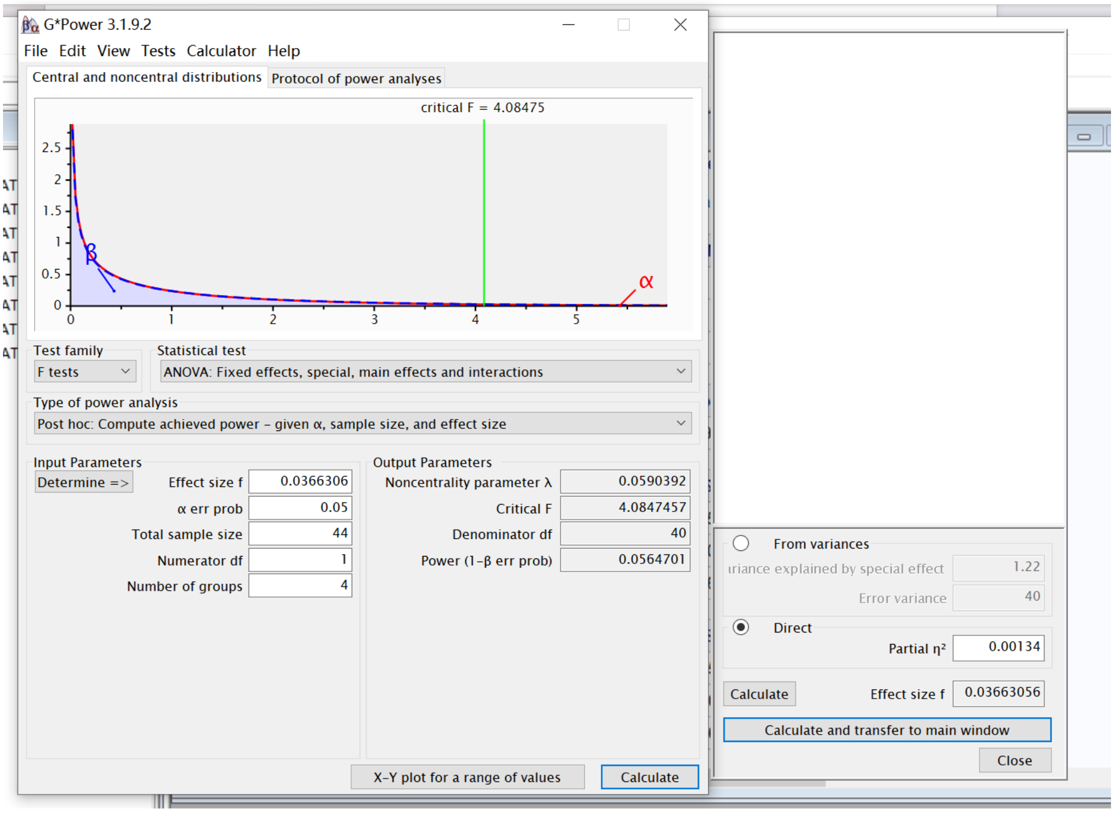

DM

A factor

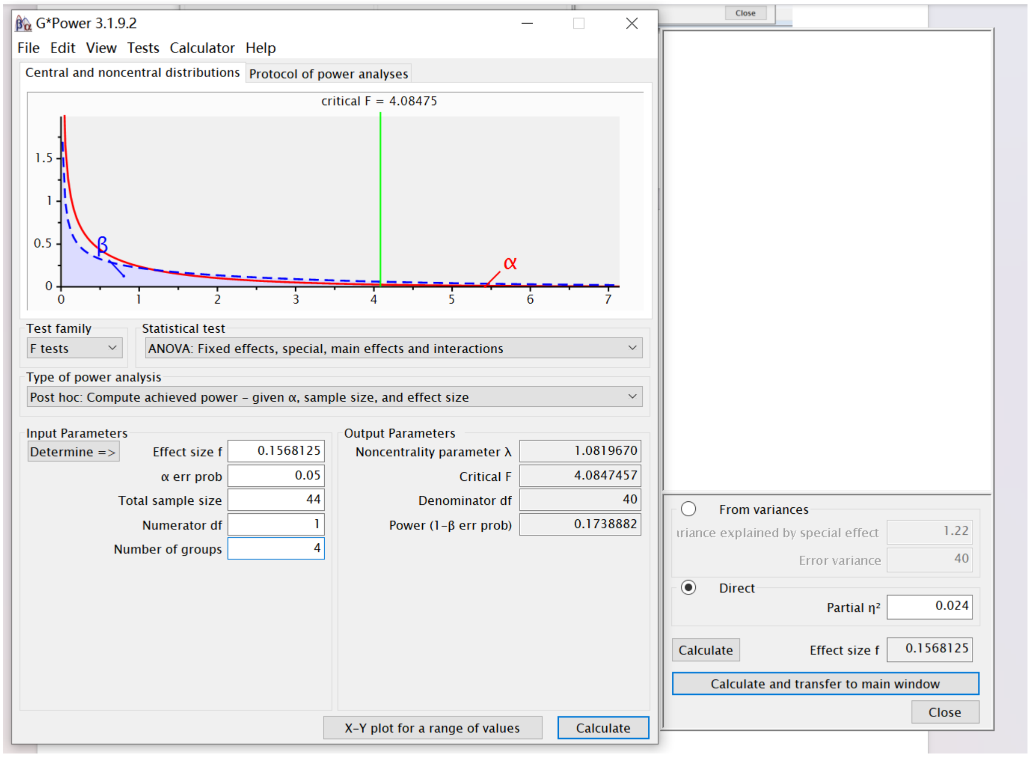

B factor

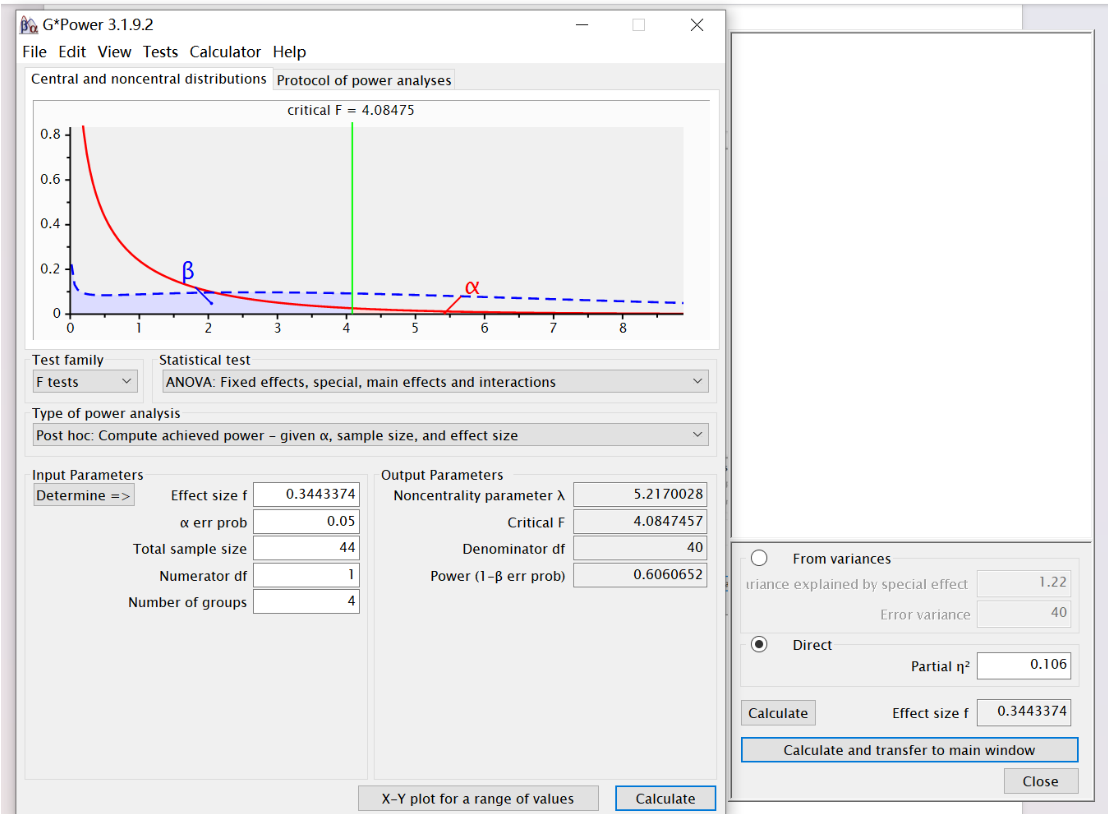
G*Power 3.1.9.2
File Edit View Tests Calculator Help
Central and noncentral distributions
Protocol of power analyses
critical F = 4.08475
Test family
F tests
Statistical test
ANOVA: Fixed effects, special, main effects and interactions
Type of power analysis
Post hoc: Compute achieved power – given α, sample size, and effect size
Input Parameters
Determine =>
Effect size f 0.3443374
α err prob 0.05
Total sample size 44
Numerator df 1
Number of groups 4
Output Parameters
Noncentrality parameter λ 5.2170028
Critical F 4.0847457
Denominator df 40
Power (1–β err prob) 0.6060652
X-Y plot for a range of values
Calculate
From variances
Error variance 40
Direct
Partial η² 0.106
Calculate
Effect size f 0.3443374
Calculate and transfer to main window
Close

A ×B

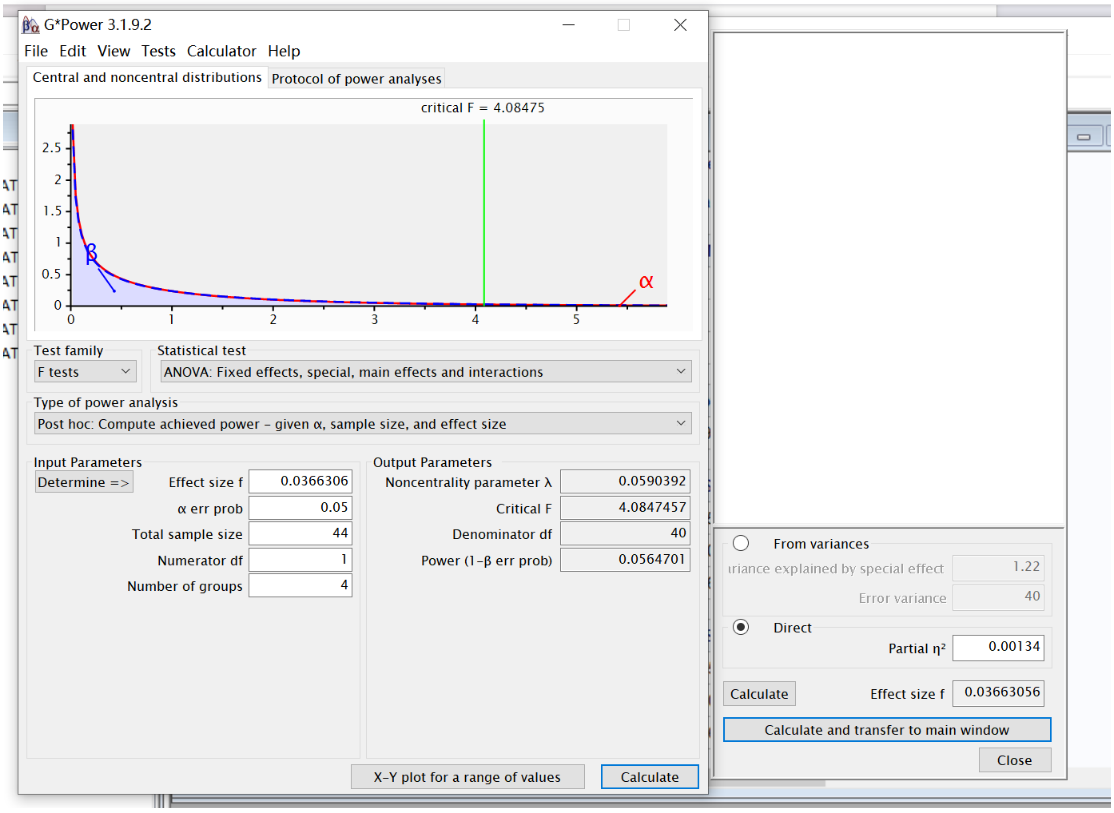
G*Power 3.1.9.2
File Edit View Tests Calculator Help
Central and noncentral distributions
Protocol of power analyses
critical F = 4.08475
Test family
F tests
Statistical test
ANOVA: Fixed effects, special, main effects and interactions
Type of power analysis
Post hoc: Compute achieved power – given α, sample size, and effect size
Input Parameters
Determine =>
Effect size f 0.0366306
α err prob 0.05
Total sample size 44
Numerator df 1
Number of groups 4
Output Parameters
Noncentrality parameter λ 0.0590392
Critical F 4.0847457
Denominator df 40
Power (1–β err prob) 0.0564701
X-Y plot for a range of values
Calculate
From variances
Error variance 40
Direct
Partial η² 0.00134
Calculate
Effect size f 0.03663056
Calculate and transfer to main window
Close

N

A factor

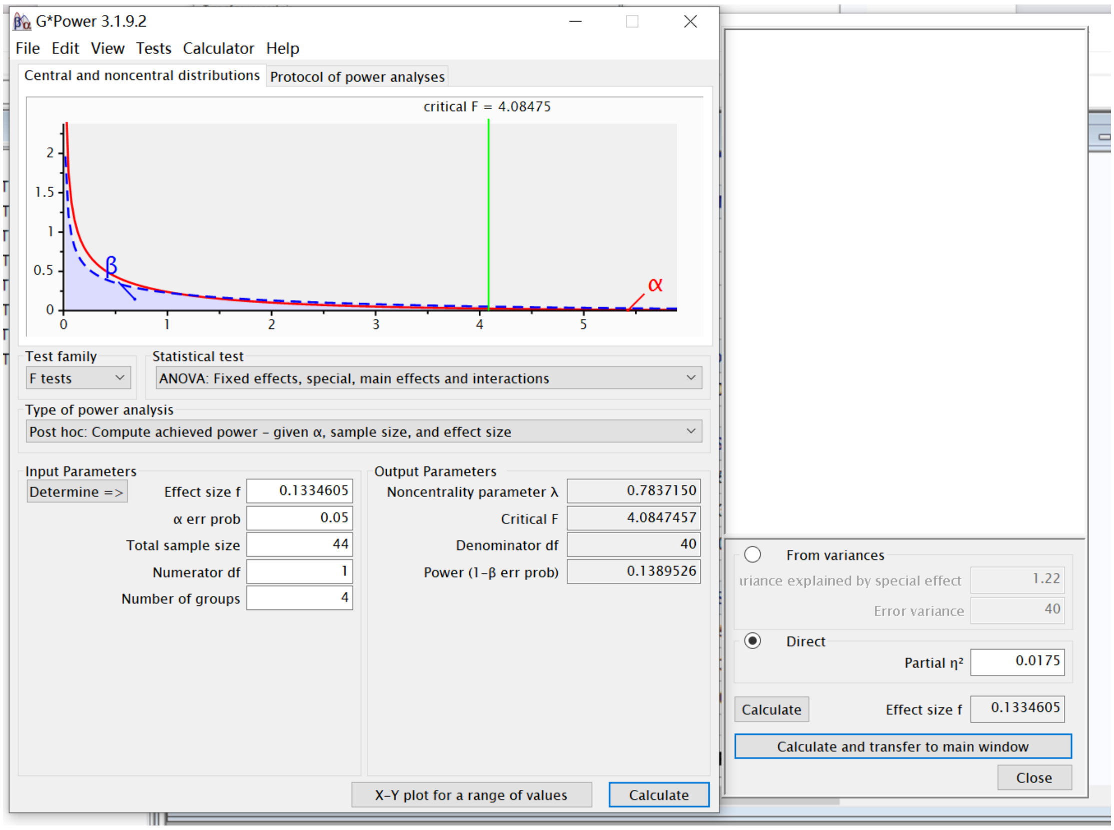

B factor

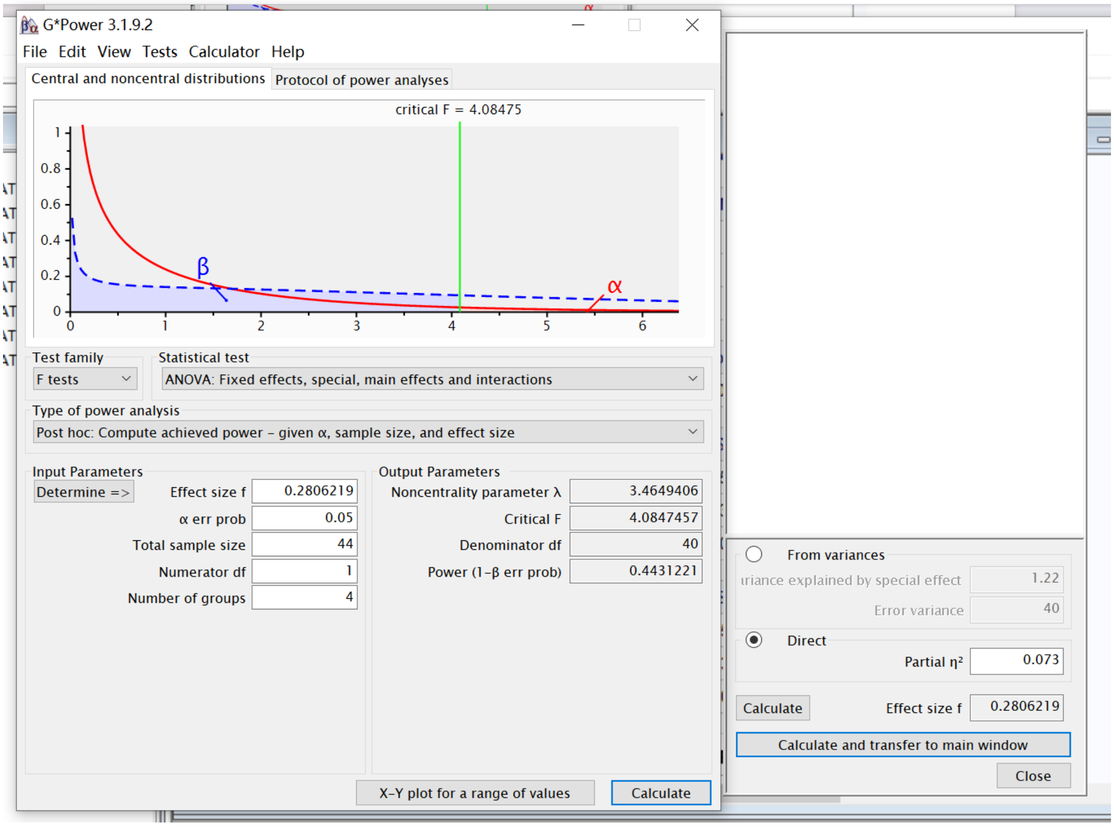

A×B

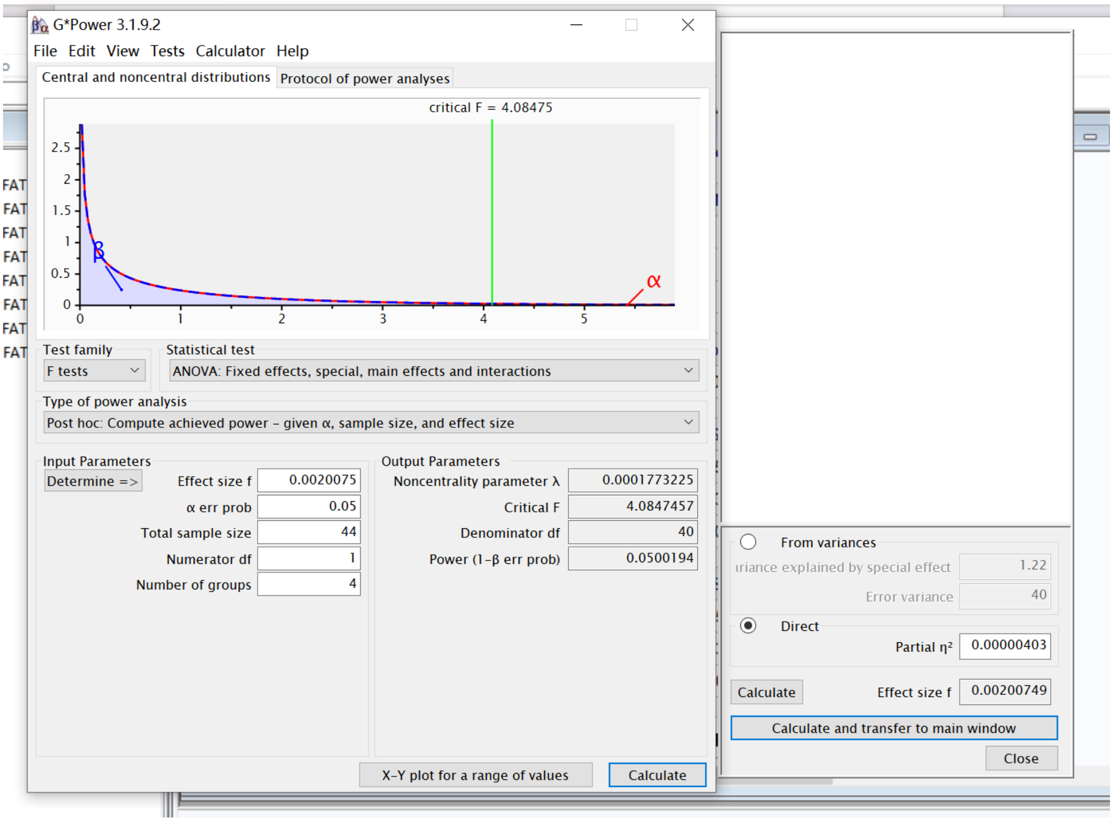
G*Power 3.1.9.2
File Edit View Tests Calculator Help
Central and noncentral distributions
Protocol of power analyses
critical F = 4.08475
Test family
F tests
Statistical test
ANOVA: Fixed effects, special, main effects and interactions
Type of power analysis
Post hoc: Compute achieved power – given α, sample size, and effect size
Input Parameters
Determine =>
Effect size f 0.0020075
α err prob 0.05
Total sample size 44
Numerator df 1
Number of groups 4
Output Parameters
Noncentrality parameter λ 0.0001773225
Critical F 4.0847457
Denominator df 40
Power (1-β err prob) 0.0500194
X-Y plot for a range of values
Calculate
From variances
Error variance 40
Direct
Partial η² 0.00000403
Calculate
Effect size f 0.00200749
Calculate and transfer to main window
Close

E

A factor

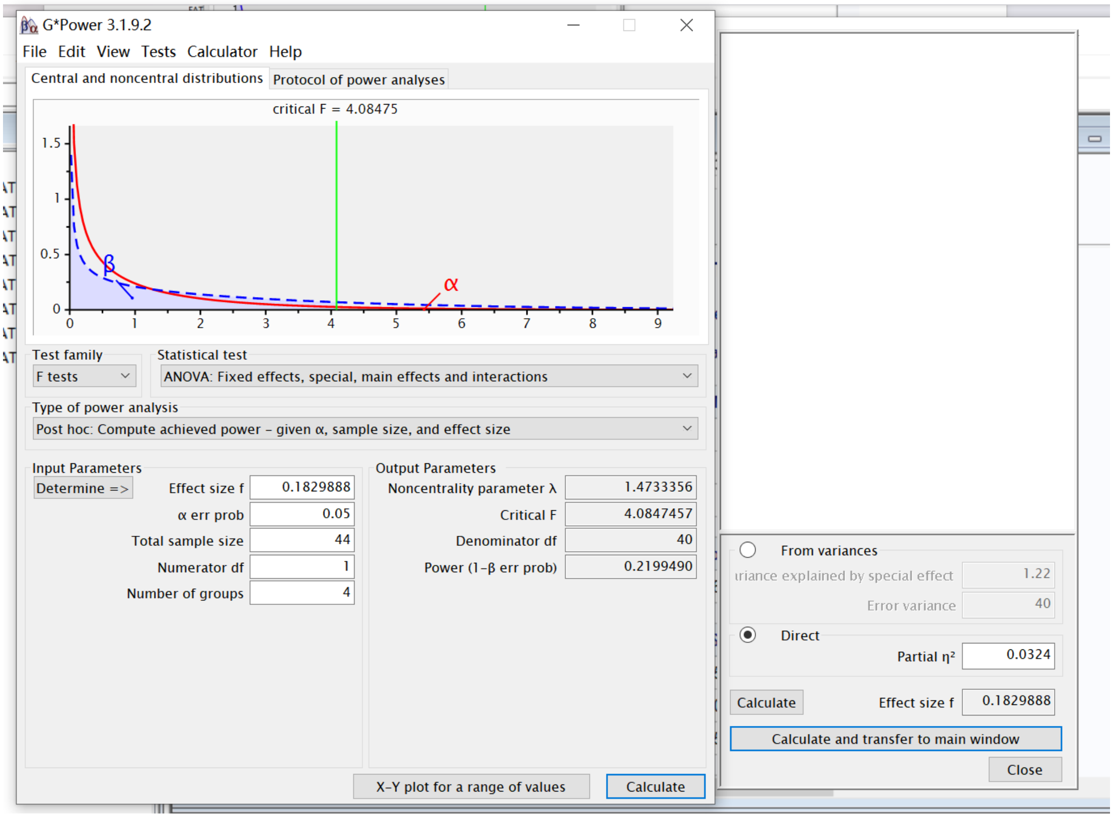
G*Power 3.1.9.2
File Edit View Tests Calculator Help
Central and noncentral distributions
Protocol of power analyses
critical F = 4.08475
Test family
F tests
Statistical test
ANOVA: Fixed effects, special, main effects and interactions
Type of power analysis
Post hoc: Compute achieved power – given α, sample size, and effect size
Input Parameters
Determine =>
Effect size f 0.1829888
α err prob 0.05
Total sample size 44
Numerator df 1
Number of groups 4
Output Parameters
Noncentrality parameter λ 1.4733356
Critical F 4.0847457
Denominator df 40
Power (1-β err prob) 0.2199490
X-Y plot for a range of values
Calculate
From variances
Error variance 40
Direct
Partial η² 0.0324
Calculate
Effect size f 0.1829888
Calculate and transfer to main window
Close

B factor

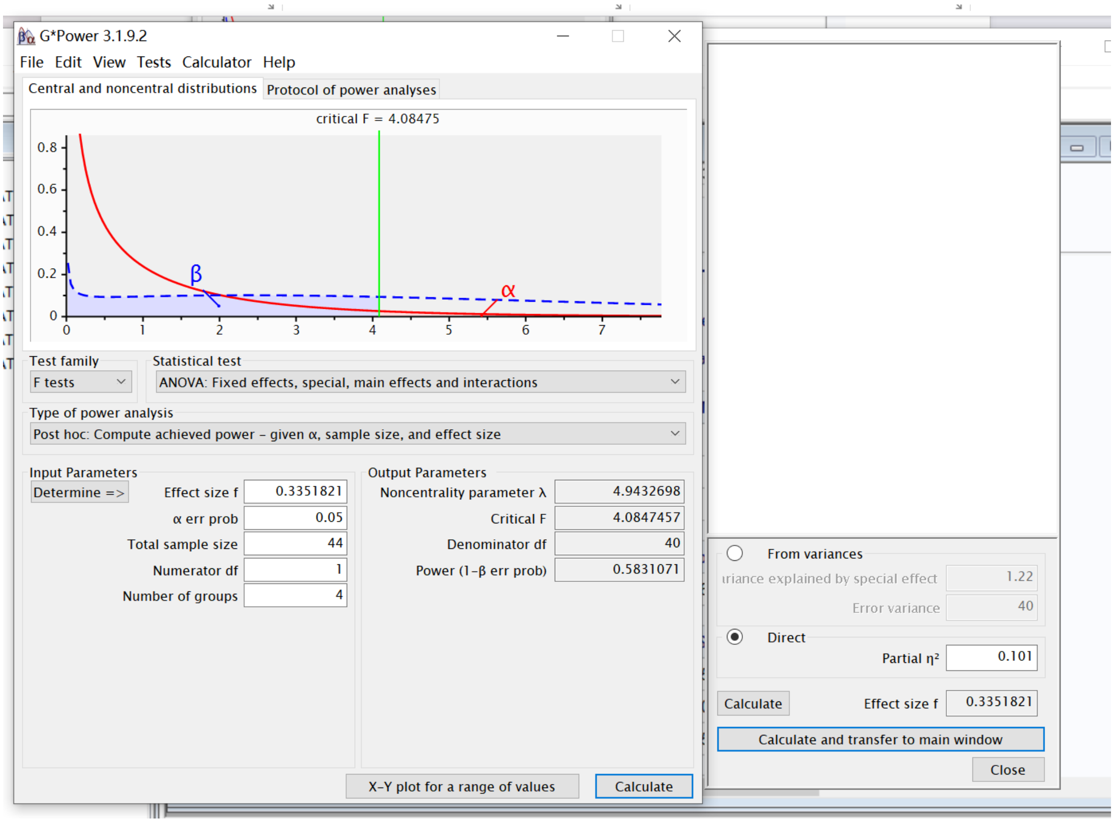
G*Power 3.1.9.2
File Edit View Tests Calculator Help
Central and noncentral distributions
Protocol of power analyses
critical F = 4.08475
Test family
F tests
Statistical test
ANOVA: Fixed effects, special, main effects and interactions
Type of power analysis
Post hoc: Compute achieved power – given α, sample size, and effect size
Input Parameters
Determine =>
Effect size f 0.3351821
α err prob 0.05
Total sample size 44
Numerator df 1
Number of groups 4
Output Parameters
Noncentrality parameter λ 4.9432698
Critical F 4.0847457
Denominator df 40
Power (1-β err prob) 0.5831071
X-Y plot for a range of values
Calculate
From variances
Variance explained by special effect 1.22
Error variance 40
Direct
Partial η² 0.101
Calculate
Effect size f 0.3351821
Calculate and transfer to main window
Close

A ×B

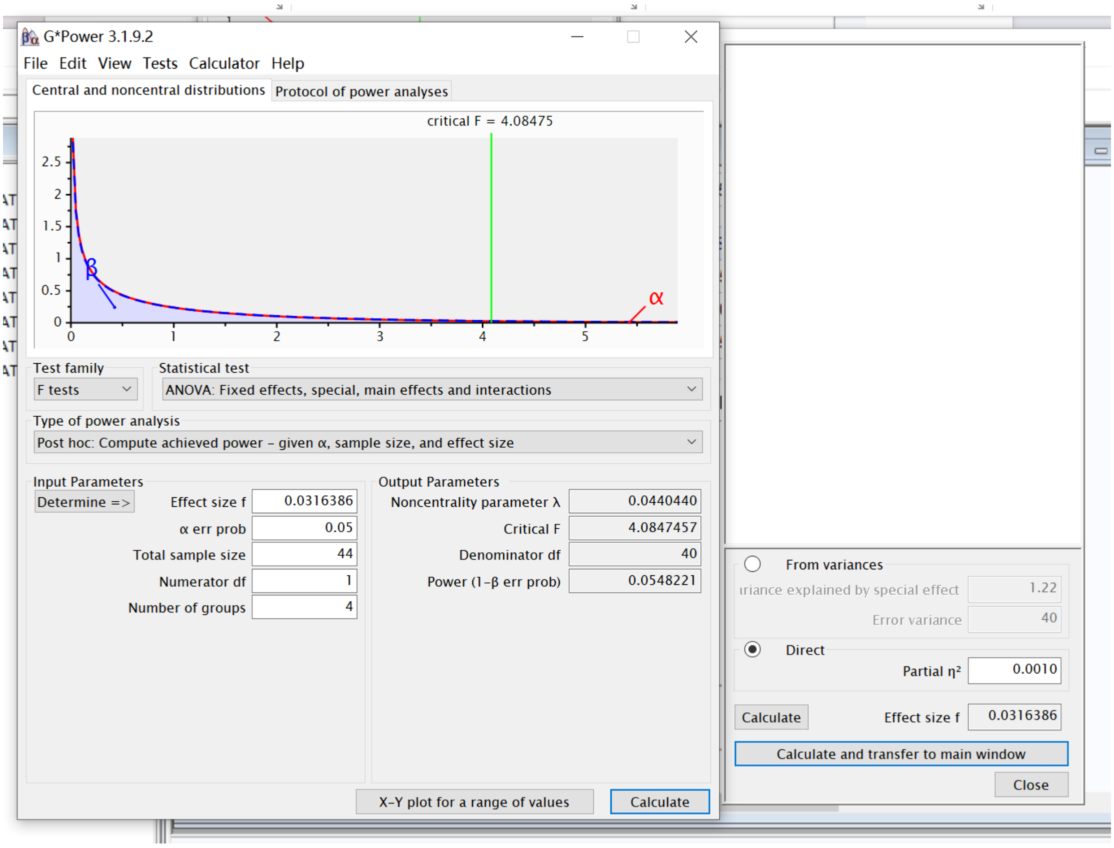
G*Power 3.1.9.2
File Edit View Tests Calculator Help
Central and noncentral distributions
Protocol of power analyses
critical F = 4.08475
Test family
F tests
Statistical test
ANOVA: Fixed effects, special, main effects and interactions
Type of power analysis
Post hoc: Compute achieved power – given α, sample size, and effect size
Input Parameters
Determine =>
Effect size f 0.0316386
α err prob 0.05
Total sample size 44
Numerator df 1
Number of groups 4
Output Parameters
Noncentrality parameter λ 0.0440440
Critical F 4.0847457
Denominator df 40
Power (1-β err prob) 0.0548221
X-Y plot for a range of values
Calculate
From variances
Variance explained by special effect 1.22
Error variance 40
Direct
Partial η² 0.0010
Calculate
Effect size f 0.0316386
Calculate and transfer to main window
Close
